# Materia Medica and Therapeutics

**Published:** 1843-01-01

**Authors:** 


### MATERIA MEDICA AND THERAPEUTICS.

I. MANUAL OF GENERAL THERAPEUTICS. By *D. Spillan*, M.D. A.M. 12mo. First Edition, 1841.

II. ELEMENTS OF MATERIA MEDICA AND PHARMACY. By *O'Brien Bellingham*, M.D. 8vo. Part I. 1841.

III. A DISPENSATORY. By *Robert Christison*, M.D. F.R.S.E. Thick 8vo. First Edition, 1842.

IV. ELEMENTS OF MATERIA MEDICA AND THERAPEUTICS. By *Jonathan Pereira*, M.D. F.R.S. L.S. Two vols. thick 8vo. Second Edition, 1842.

### I. A MANUAL OF GENERAL THERAPEUTICS.

WE have much pleasure in introducing to our readers a very useful little work on General Therapeutics—on the *general* actions, physiological and therapeutical, of medicinal agents. In works on Materia Medica, we usually have an Introduction on the "*General Actions of Medicines;*" and if the therapeutical arrangement has been adopted, we have general remarks also, on each *class* of medicines; general remarks, for example, on purgatives; on narcotics, &c. Our readers then are to imagine these observations enlarged, brought together, and collected into one volume, and they will form a very tolerable idea of the work under consideration.

In the first Chapter the nature of therapeutics is defined, and the object of the author, in the present volume, set forth. "By Therapeutics is meant that department of medical science which treats of the cure or palliation of disease." And although it must be confessed, that the treatment of disease is at times empirical, yet our author hopes that, as the functions of life become more investigated, the action of medicines will become more understood, and the treatment more rational. Many preliminary sciences are necessarily known, before the study of therapeutics can be successfully pursued.

"Physiology observes man in the healthy state, and traces the laws according to which the several functions of the body are performed. Pathology directs attention to the causes which change the structure and derange the functions of the different organs, investigates the nature of such changes and derangements, and observes the phenomena accompanying them. The science of Therapeutics next comes in, and, guided by the knowledge previously derived from physiology and pathology, it establishes the various indications to be fulfilled, and determines the method necessary for restoring the system to its healthy state. The insufficiency of a knowledge of mere symptoms is now acknowledged on all hands. Diseases are now known to depend on lesions of certain tissues or organs. The modern therapeutist, directed by the lights of modern pathology, studies the nature, character, and product of these lesions, and considers the symptoms no farther than as enabling him to arrive at a knowledge of the seat of the pathological lesion producing them, and the structural alteration of which they are but the expression. The lesions, however, sought by the therapeutist, are not exactly those presented by the dead body; such lesions have reached their final termination, and have passed the limits within which the resources of medicine could effect any good. We must be aware, therefore, that in the lesions found in the dead body, many pathological elements have disappeared which kept up several of the symptoms, both direct and sympathetic, in the relief of which medical treatment may have been available. Death has now substituted icy coldness for those exaltations of temperature observable in several parts of the living body, and the cause of so much distress to the patient; paleness has now taken the place of inflammatory redness; where there was tension, there is now absolute laxity of parts. Several pathological phenomena observable on the living body, and which might have claimed the attention of the practitioner, and furnished him with useful indications, are now gone. Thus, then, it is not alone the lesions found on the dead body, but those also revealed by the symptoms, and accounted for by the laws of physiology and pathology, which the therapeutist should study, as it is these he is called on to treat." 4.

But the lesions, and all the symptoms which result from them, may be known, and yet the physician be far from a successful practitioner. It is the aggression of disease, and incipient functional disturbance that he has frequently to treat. He should be acquainted, consequently, with the power of remedies, with what they really can do, and also with the power which is justly attributable to the vis naturæ.

Dr. Spillan concludes this chapter, which is strictly introductory, with allusion to this vis naturæ.

"There has been observed in every living body, as well in the vegetable as in the animal kingdom, an instinctive principle, the constant tendency of which is to preserve the body in the healthy state by repelling the action of morbific causes, and to restore the same body, when injured by external violence, or by causes existing within itself, to its pristine state of health and vigour." "It is by the aid of this instinctive power or principle that we so frequently observe diseases removed without the intervention of art, or without even the slightest change being made in those external influences which may be supposed to have occasioned the disease; we often see diseases cured under the most opposite modes of treatment." "It constituted the *anima* of Stahl, the *archæus* of Vanhelmont; and though the moderns are not so much disposed as their predecessors to embody the refined abstractions of metaphysical philosophy, there is some reason for thinking that this same principle is still recognised in practical medicine, *mutato nomine,* under the new designation of *re-action.*" 7.

Our author now expresses a caution against placing too much confidence in this vis medicatrix on the one hand, or being too disregardless of it on the other, and he concludes—

"Thus, when in acute disease we observe signs indicating salutary efforts, as, for instance, a displacement of the irritation to the kidneys, integuments, or to the intestinal canal, the judicious and experienced physician will at once see that he has nothing else to do but to favour these movements. If the symptoms of disease are slight, and the evil is likely to disappear spontaneously, we must, of course, abstain from active treatment—yet in such cases the physician is not really passive; he observes the phenomena as they present themselves, and modifies the animal organism by carefully directing and judiciously favouring the tendency, ascertained by him, to the re-establishment of health." 9.

CHAP. II.—We now proceed more particularly to our subject. We are writing on the treatment of disease. What then is an indication for treatment? Indications in disease for treatment are of several kinds; they occupy this and the next chapter. The indications of the present chapter are either *empirical, rational, fundamental, occasional,* or *symptomatic.* The term "fundamental," as applied to an indication, is new to us; we transcribe our author's exhibition of it.

"By fundamental indications, those are understood which are derived immediately from the exact knowledge of the nature, causes, and course of the disease. For instance, a lady, of thirty-six years of age, suddenly meets a gentleman for whom she entertained a very ardent affection, and whom she had not seen for a very considerable time; the unexpected meeting produces a powerful effect on her, and causes a suppression of the menses, which happened to be flowing at the time. Some time after the shock, &c. she fainted; when visited by her physician on the next day, her pulse was full and frequent, her face was flushed, skin hot, every object in her chamber seemed of a red colour; her mind was a good deal disturbed, and violent spasmodic and convulsive movements were observed to occur: in this case the obvious and fundamental indication was to draw blood, which, being followed by sedatives, soon restored the patient to the healthy state." 12.

The others need no comment.

CHAP. III. contains the Therapeutic Indications derived from Disease. It is divided into two sections.

1st. Therapeutic signs derived from the morbid phenomena of the organs or systems of organic life.

2nd. Therapeutic indications derived from the morbid phenomena of the organs of animal life.

Under the first head are the following:—desire of food, thirst, vomiting, constipation, diarrhœa, defæcation, the pulse, respiration, fætor of the breath, animal heat, sanguineous exhalations, urine.

These are stated clearly, and at some length; and their general value set forth, as well as their value in particular cases. Vomiting may serve as an instance.

"*Vomiting.*—When this is the sign of gastric irritation, of cancer of the stomach, or of strangulated hernia, we must of course avoid making the patient vomit, lest we aggravate his sufferings, and even occasion death.

"When, however, vomiting is the result of simple indigestion, unaccompanied by any previous irritation, it may be advisable to promote it by means of tepid water; diluent drinks, and strict attention to diet, are then sufficient to effect a cure.

"It may be stated that vomiting will possess a very different therapeutic value, when it depends on the diseases just now mentioned, and which all have their seat in the stomach, or on a disease of some remote viscus, such as the brain. It is almost unnecessary to remark that the quality of the substance vomited, as it frequently indicates the nature of the disease which occasions it, will often furnish correct diagnostic signs; it is obvious that the vomiting of blood and of simple alimentary substances admits not of the same mode of treatment.

"From the changes which occur in the functions of the intestinal tube, and in the quality of the fæces, some therapeutic indications may be derived; such signs, however, are of little value when unaccompanied by other signs." 23.

Under the second head, Dr. S. includes such indications as attitude of the body, œdema and anasarca of extremities, state of the skin, its colour, its dryness, the expression of the countenance, the character of the pupil, or depression of muscular power, peculiar alterations in the voice, as in maniacs, general sensibility of the body, as in hysteria, and lastly—*pain.*

With reference to pain, says our author :—

"The question presents itself, whether the pain which accompanies the great majority of diseases, and which presents in each a peculiar character, can be combated by particular means? Those practitioners whose leading theory it was that each individual symptom required to be met by a special remedy, and who, in accordance with this theory, employed opium to relieve inflammatory pain, would answer this question in the affirmative. And though such a therapeutical principle is anything but sound, still every medical man knows that there are cases where he is obliged to regulate his conduct in conformity with it. Thus, in the case of violent pain, a special remedial agent may be directed to combat it; 1st, in the case where it constitutes the principal phenomenon of the disease, as in nervous pains; 2nd, when in chronic diseases the pain becomes very violent, and narcotics, employed internally and externally, cannot increase the principal disease. In other cases, however, it is the disease, of which the pain is but the companion, that we must combat.

"The nature also of the pain may render it necessary to modify the treatment. If the quality of the pain denotes the existence of suppuration, blood-letting must be suspended; which must again be resorted to, if the pain indicate sanguineous congestion, or acute inflammation. In a word, our curative agents must vary with the nature of the disease characterized by the pain. It is unnecessary to say that the pain of inflammation, nervous pain, the pain of rheumatism or of gout, that which depends on intestinal gases, on accumulated fæces, worms, &c. should not be treated after the same manner; another proof, if such were wanting, that all rational and judicious medical treatment must depend on the accuracy of our diagnosis." 38.

Dr. S. now mentions the *therapeutic indications* derived from the *morbid phenomena of the genital organs and functions,* referring especially to the female system. He shews the importance of regularity in the menstrual flux; and the various vicarious diseases which may depend on its suppression. He also alludes to menorrhagia and dysmenorrhœa, as symptoms.

The *causes of disease* are next considered as therapeutical indications.

Thus *colic* may be produced by many causes; but if "a patient complains of severe colic, and we find that his occupation obliges him to continually handle lead, we are at once led from a knowledge of the cause to select the appropriate treatment."

The seat of the disease furnishes most important therapeutic indications. Dr. S. observes:—

"When the physician is called to a patient, his first duty is to discover what organ is the seat of disease. All his researches into the history of the case, all his investigations with respect to the disturbance of the functions, are solely to enable him to ascend to the knowledge of the parts primarily affected. We cannot have a just and complete idea of a disease, until, after having ascertained its nature and the mode of action of the causes which have excited it, we are able to trace the impression of these causes on one or more organs, and to determine the connexion of the lesions and of the phenomena which have been successively developed. It is only then the physician can deduce the curative indications arising from such a state, and the most effectual treatment to be employed; for it is then only he is able to trace back in thought the various modifications which the organs must undergo in order to be restored to their healthy state.

"Without accurately knowing the parts affected, how should we apply the appropriate treatment to paralysis and convulsive affections, which are the effect of the primary or secondary irritations seated in the brain and its membranes? The fundamental basis of therapeutics, as well as of pathology, obviously consists in the knowledge of the seat and nature of diseases." 50.

The chapter is concluded with the indications drawn from the course, duration, and period of diseases.

"Every practical physician must be aware of the great influence which the periods or stages of disease should exercise on the choice and activity of the remedies to be employed. Thus we often witness the extraordinary good effects of blood-letting at the very commencement of inflammation; a remedial agent, which, though often necessary in the advanced stage of the disease, is never observed to produce such striking and almost marvellously good results as at its onset. Again, when the inflammation has ended in suppuration, even though the fever may still continue very high, blood-letting is but rarely attended with advantage." 52.

Having thus shewn the various indications of treatment, we are led naturally, in Chap. IV. to

### CIRCUMSTANCES MODIFYING THESE INDICATIONS.

The age and sex much influence the indications for different remedies. A different treatment is required for the same disease, in persons of different professions, and with peculiar habits. The temperaments, also, of the patient must be taken into consideration, and any peculiar idiosyncracies. Further remarks on these points would be superfluous; we pass at once to the next, a very interesting chapter.

### ON THE MODUS OPERANDI OF MEDICINES.

Dr. Spillan commences this chapter with noticing the general properties of medicines.

"Every medicinal substance possesses a peculiar power, which may be explained either by its physical or chemical qualities, and which is only developed on being incorporated with the living organism. When the medicinal substance and organism come together, there occurs in the latter a series of changes and phenomena which go by the name of medicinal action." 68.

These changes may be either local, or, what is termed, sympathetic. If the seeds of aconite be chewed, they produce a peculiar tingling of the lips and tongue—this is their local action. The serious symptoms which result from swallowing many of these seeds, is sympathetic. This term sympathetic, is a bugbear term, a giant monster, which every reader of therapeutics dreads, at first sight. We are glad to find it fully discussed. "The question now presents itself (says our author) how can a medicinal substance exert an influence on parts remote from the place of application? The common and very generally prevailing opinion is, that the nerves transfer the local impression made by the action of the medicinal substance to remote parts and organs. According to this view, the remote actions of medicines are divided: 1, Into those which may be accounted for by the consensus of the nerves. 2, Into those actions which are occasioned by proximity of parts."

A local action in both cases is here made a requisite; and the secondary effect is induced by transmission of the primary action to distant parts of nervous communication, or the proximity of parts. Dr. S. conceives that some agents do act so. Purgatives for example. But the action of others he considers to be totally inexplicable on these grounds. "We may further satisfy ourselves that we can, for the most part, explain the remote effects only by admitting that a material action of the medicinal substance passes to remote organs from the point of application. But such a material action can only take place by the medicinal substances entering into the circulation."

Dr. S. now urges some objections to the theory of secondary action by nervous impression, and proceeds to the substantiation of the theory of absorption. He lays down the following fifteen rules for assuming the *entrance* of substances into the blood.

"1. If medicinal substances are found in the blood.

"2. If medicines are found in the secretions and excretions.

"3. If the substances employed are found deposited in the solid parts of the body.

"4. When, after the application of a medicinal substance, the effects of the same on remote organs do not follow till a late period, and in every case perceptibly later than the local effects. Action of narcotics is given as an instance.

"5. When, on the application of a medicinal substance, neither structural nor functional changes occur at the place of application, but on the contrary, considerable effects are produced on remote organs.

"6. When a medicinal substance, no matter how various the forms and ways in which it may be applied, always produce the same phenomena.

"7. When one and the same medicinal substance given in the same dose does not produce stronger remote effects proportioned to the importance of the organ to which it may be applied.

"8. When one and the same medicinal substance produces sometimes greater, sometimes less effects at the place of application, and when these do not produce corresponding remote effects; that is, when intense local effects are not followed by equally intense phenomena in remote organs, but, on the contrary, the more intense the remote effects are, the less the local symptoms are found to be.

"9. When a medicinal substance, applied to a part or organ whose nervous connexion with the rest of the body is cut off, still exerts its influence on this, and with it also on the central parts of the nervous system.

"10. When the nervous communications continuing perfectly entire, but the circulation of the blood being cut off in a part of the body, a medicinal substance applied to this part produces no action on remote organs.

"11. When a medicinal substance, after local application to a wound, produces no action on remote organs, so long as its absorption is impeded by any means, but on the other hand acts, if nothing interferes with its absorption.

"12. When the activity of a medicinal substance is diminished, nay perfectly destroyed, by an artificial plethora.

"13. When a substance applied to parts not furnished with nerves, yet produces effects in distant organs, even when locally, no change of structure whatever is observed.

"14. When medicinal substances introduced immediately into the blood act in just the same way as when they are taken internally, or exhibited in any other way.

"15. When a medicinal substance gradually disappears at the place of application, that is, is absorbed, and occasions in distant organs effects which correspond with the disappearance of the medicinal body." 83.

But, as Dr. Spillan continues to remark, *some* remote actions are without doubt attributable to local action and nervous conduction, as diffusible stimuli: and having just given instances in which remote actions were dependent on absorption, he now shews us others which are due to local action and nervous sympathy.

"We may," says Dr. S. "safely lay down the following rule:

"When a medicinal substance has produced considerable local changes of structure or function, sympathetic effects, to a greater or less extent, must also necessarily be produced in remote organs." 87.

And the question now is, to distinguish between the remote effects from absorption and those from nervous sympathy. The following limitations attend the operation of the latter cause; and the actions to which they are inapplicable must be considered due to absorption.

"1. When the remote action of a medicinal substance is a sympathetic one, this must correspond in strength with the local action."

"2. The remote action, in case the nerves are the means of conduction, becomes so much the more intense, the more important the organ is which is locally affected by the medicine."

"3. A sympathetic action, which would manifest itself in a remote organ, may be in part at least avoided, by cutting off the nervous connexion between the place to which the substance is applied, and the rest of the body."

"4. The remote effects occasioned by consensus of the nerves, and which are solely the consequence of the local medicinal action, follow always immediately after the local action, cannot take place without this, are not prevented by an artificial plethora, &c." 89.

Granted, then, that many medicinal substances do not produce their effects until they have become mixed with the blood, the next point of enquiry is, how they "make their way into the torrent of the circulation." Dr. S. considers that they enter *principally* by the veins; by direct imbibition through their coats. We can hardly curtail the following remarks:—

"Experiments the most satisfactory have proved that medicinal substances may be taken up into the circulation from the entire surface of the body, from the entire tract of the intestinal canal, from the mucous membranes of the air-passages, &c. &c. It is also known that the coats of the blood-vessels are pervious to medicinal substances; but whether these substances are taken up mediately through the lymphatic vessels, or immediately through the blood-vessels, and so brought into the blood, or whether they reach the circulation in both ways, is not so clearly made out.

"In determining this matter, it should be kept steadily in view that the question is, whether medicinal, and therefore heterogeneous, unassimilable substances, but not whether all, and therefore assimilable matters, are brought into the blood by the lymphatic vessels, or by the blood-vessels. It is certain that the latter (assimilable substances) are carried into the blood exclusively by the lymphatics, and that chyle has been but very rarely found in the veins of the intestinal canal. No inference, however, can be drawn from this regarding the relation subsisting between the absorbents and medicinal substances. From repeated experiments, instituted for the purpose of determining this question, the following inferences have been made. In support of the absorbing power of the blood-vessels, with respect to heterogeneous medicinal substances, we have:—

"1. The observation that medicinal substances enter the blood on coming in contact with the parietes of the blood-vessels.

"2. In support of the absorbing power of the blood-vessels, and against that of the lymphatics, with respect to heterogeneous substances, we may adduce the more frequent occurrence of the latter in the blood, and their very rare appearance in the lymph and chyle.

"3. In support of the absorbing power of the blood-vessels, we have the experiment that absorption takes place in parts of the body which possess no absorbents.

"4. For the absorbing power of the blood-vessels, and against that of the lymphatics, we may adduce the rapidity with which certain substances enter the blood.

"Wherever a medicinal substance is applied, whether to the air-tubes and bronchi, or to the surface of the body, after they have penetrated the non-vascular epidermis or epithelium, they meet with a fine, delicate network of blood-vessels, diffused over every part, which presents to medicinal substances a much greater surface than the lymphatic vessels do in any part. The probability that any considerable quantity of those substances is taken up by the absorbents, would accordingly be rendered extremely doubtful on these grounds; and still more so, if these vessels, as is not unlikely, have the power of absorbing only by their roots, but not of admitting such substances to pass through their coats.

"But supposing the parietes of those vessels were as pervious to medicinal substances as those of the blood-vessels, still the former will not only bring a much smaller quantity of them into the blood than the blood-vessels, because they present a smaller surface to the medicinal substance, but also on this account, because the fluid contained in them reaches the blood very tardily. It is not sufficient that medicinal substances should penetrate a vessel, but when they have entered it, it is necessary that they be carried on with rapidity, in order that they may act on the entire body. This property of rapid conveyance we know the blood-vessels possess. Thus Mayer found ferro-prussiate of potash in the blood two or three minutes after a solution of it was injected into the air-tube of an animal. Westrumb found iodine in the urine of a dog ten minutes after its internal exhibition. In the case of a boy with congenital prolapsus vesicæ, Stehberger, after administering internally madder and tincture of indigo, found them in the urine after fifteen minutes, as also rhubarb and gallic acid after twenty minutes, by means of the colour and by re-agents.

" If we now compare this rapid passage of medicinal substances into the blood with the tardy course of the lymph and chyle, we will at once see that the former cannot reach the blood in this way." 92.

Having considered the way in which medicinal agents get into the blood, our author now proceeds to grapple with these all-important but difficult questions.

1. How the blood and medicinal agents re-act on each other, when they meet.

2. How the blood thus charged with the new substance re-acts on the nervous system by which the secondary effects are produced.

With reference to the first proposition—are they merely mixed or do they enter into chemical union with the blood ? Probably both positions are true, though of different medicines. Thus, the volatile oils are only mixed ; but if any one seeks for *mercury* in the blood, he will be obliged to decompose it entirely. It seems, like iron and sulphur, to be in a state of primary combination with the ultimate elements of the blood.

With reference to the 2nd proposition—Dr. Spillan says,—

" If we suppose a medicinal substance circulating in the blood, the first question which forces itself on us is, how does this act on the parietes of the vessels ? Considering that medicinal action can take place only where there are nerves, we must conclude, *à priori,* that in the larger trunks, and even in the smaller vessels, no medicinal actions go on, there being no nerves on the inner membrane of these vessels. Medicinal substances which enter the circulation can, therefore, only act there, where all change of substance and all reciprocal action between the solids and fluids take place, namely, in the capillary vascular system. But it is only the central parts and the peripheric terminations of the nervous system that are in the most intimate and multiplied contact with the capillary system." 98.

But this is rather the "*where*" than the "*how*" medicinal agents act on the nervous system This "*how,*" we think, cannot be understood until we thoroughly comprehend how the blood itself acts on the nervous system—how, in fact, the nervous energy is itself secreted.

The chapter is concluded with a consideration of what may be termed the *elective action of medicines.*

Dr. S. continues,—

" Though there are entire classes of medicines which influence all the organs with tolerable uniformity, still, on the other hand, it is not to be denied that the most important medicinal substances exert, if not their exclusive, at least their principal action on certain individual organs. Thus nux vomica exerts its chief action on the spinal chord, opium principally affects the brain, digitalis and tobacco the heart, cantharides the genital and urinary organs; in short, the most important medicinal substances act specifically on certain individual organs." 104.

It is certainly difficult to assign the cause of such results. Our author is of opinion, that the different organs have what he terms, " a special susceptibility for certain medicinal agents," and he supports this position by the following facts.

" 1. Particular medicines occasion an increased afflux of blood in the organs on which they chiefly exert their influence, and even excite in them structural changes." 105.

As an illustration of this position, Dr. S. quotes the experiments of Flourens on opium and belladonna. The latter acts, according to Flourens, on no part of the brain but the corpora quadrigemina, and consequently causes dilatation of the pupil. This *may* be true, but we call it in much question. Opium, according to Flourens, acts principally on the lobes of the brain; "an inference deduced from the observation, that after poisoning with opium those parts are always the seat of sanguineous effusion, the other parts of the brain being entirely unchanged." We cannot coincide with M. Flourens here; *any one*, we think, will admit, not only *cerebral* congestion as a result of poisoning by opium, but venous congestion universally.

"2. Medicines which act on particular organs are occasionally deposited in them."

"3. Medicines which promote certain secretions are excreted with them." 105.

The number of diuretic substances detected in the urine is adduced in proof of this last proposition. The processes of nutrition and secretion are considered to be analogous in the elective affinity of the different organs for the elements of which their secretions are composed. With reference to nutrition, Dr. Spillan observes,—

"It is certain that the proximate principles of the organs partly exist already in the blood; such as the fibrine, albumen, &c. These several substances, in contributing to nutrition, are not deposited accidentally here and there, but they are attracted by similar organic parts; thus fibrine is attracted by the muscles, albumen by the glands, &c. There is then, during the process of nutrition, a species of elective attraction between certain organs and certain substances contained in the blood, which bears at least some resemblance to the process that takes place when certain medicinal substances are deposited only in those organs on which they are destined more immediately to act." 107.

Also, with respect to the secretions,

"It has been proved that many of them are already present in the blood. Now we know that these are usually eliminated from the body, each by some particular secreting organ; a fact which can only be explained by admitting that these secretions already formed in the blood are more especially attracted by certain organs." 107.

The elimination of urea from the blood by the kidneys is a fair illustration; it is due to their elective attraction for that substance.

"As a further proof of such attraction, (he continues,) it has been ascertained to possess diuretic properties, whether taken internally, or injected into the veins. Vauquelin and Segalas, Fouquier and Laennec, have given it accordingly as a medicine. It is well known that the Arabians used the urine of the sheep and of the ass as a diuretic." 108.

Dr. Spillan concludes thus:—

"All these phenomena are accounted for by considering that every organ with its nerves forms a more or less independent whole, possessing its own peculiar vitality and formation. All the organs must therefore manifest a different sensibility or impressibility towards external or relatively external stimuli, according to its peculiar endowment. This is illustrated in the process of nutrition, and in the several secretions, as well as in the functions of the organs of sense, and the case is precisely the same with respect to substances circulating in the blood which produce different effects in different organs." 109.

We need only mention the subject of the next chapter. It is "the surfaces adapted for the application of medicines," and we pass at once to Chap. VII. "On the Effects of Medicinal Substances."

The *effects* of medicines are described by Dr. Spillan as primary and secondary, physiological and therapeutical. He thus distinguishes them.

"The action of a medicinal substance, taken in the proper dose, may be distinguished into two periods, or divided into two parts. 1st, Its contact with the organs of the body calls forth the development of its active power; the latter is instantaneously put forth, and certain changes in the state of the surface to which it is applied denote its power. In a little time—whether the molecules of the medicinal substance enter into the circulation, and are thus distributed over the system by the blood, or whether sympathetic communications transmit to other parts the impression first made on this surface—certain general effects are observed to supervene; the several organic tissues no longer continue in the same state, the several vital acts follow a different rhythm, all the organs take on another order of movements. These changes, occasioned by the direct impression of the medicament on the living parts of the body, constitute the first period of its action, and may be called its *immediate* or *physiological effects*. 2nd. Those changes in the actual state of the organs, those modifications in their movements, the new direction given to their vital functions, may effect some important result in a body morbidly affected; they may oppose, diminish, and combat pathological lesions, and arrest their progress; they may excite organic efforts, which will prove salutary; the disease may abate in violence and intensity, and a marked improvement in the condition of the patient may be obtained. Such a result will constitute the second part of the effects of this medicine, and are generally called the *secondary* or *therapeutical* effects." 127.

Hence the secondary or therapeutical effect is consequent on the primary or physiological; and as the minute organization of all persons is somewhat different, the variable effect of remedies is accounted for.

Dr. S. observes very justly,—

"The immediate or primary effects of a medicine are always sure to take place, whenever such medicine is employed; if any difference should occur, it will be in degree, not in kind. Thus, an excitant will always stimulate tissues; purgatives will always irritate the intestinal surface; but the curative effects are not thus constant and uniform."

The *therapeutic* effects will depend on many contingent circumstances.

The subject of Chap. VIII. has almost been anticipated from the above conclusions. This chapter treats of the "*therapeutical action of medicines.*" Dr. S. lays down and establishes the following proposion, that,—

"Medicines possess no special power distinct from their physiological action, and to which we can attribute the curative effects following their employment." 139.

We are now brought to the classification of remedies in Chap. IX.—a point of no small difficulty. Dr. S. mentions these methods—that of Barbier—Murray—and that of Dr. Billing.

Dr. Murray's therapeutical classification is well known. Dr. Billing has proposed a classification of medicines founded on their physiological action. He makes but four great classes—*stimulants, sedatives, narcotics,* and *tonics.* But these are sweeping classes. For example, purgatives, emetics, nauseants, refrigerants, bloodletting, &c. &c. are all included under sedatives, for they all ultimately produce diminished action of the heart and arteries.

In the present work, Dr. Spillan offers no peculiar classification of his own. He observes,—

"In the classification which I shall adopt in this work, both in that part which treats of the physiological effects and therapeutical employment of the several classes of medicines, as also in the formulæ appended to the book, I shall endeavour, as far as I can, to preserve the spirit of Dr. Billing's arrangement; and, accordingly, whilst I adopt the four grand classes of narcotics, stimulants, sedatives, and tonics, I shall further present the therapeutical uses of the various subdivisions of medicinal agents, as found in the generality of works on Therapeutics." 152.

In the three remaining chapters Dr. S. enters fully into the history of the classes of medicines arranged according to their therapeutic action, —narcotics, stimulants, tonics, astringents, purgatives, emetics, diuretics, &c. &c. He defines each class according to their physiological effect; and shews how this effect is produced, which is always, in reality, by one of the four methods mentioned by Dr. Billing. He points out the organs more particularly affected by the secondary action of the different classes of medicines, the indications for their exhibition, and lastly, their therapeutical application. We need only take one example. Under the head of stimulants, Dr. S. remarks:—

"Stimulants may be defined to be 'agents which increase the organic actions by impressing the contractility of the part to which they are applied, the stimulation so induced being extended, or not, to the rest of the system.'" 194.

"Most of the stimulating agents used in medicine are obtained from the vegetable kingdom; the stimulant property depending on the presence of an essential oil, or some similar principle; this oil is usually separated by distillation from the plant, as in the case of carraway, aniseed, lavender, &c.

"The *general effects* of stimulants are an exaltation of the functions of innervation, circulation, and calorification; thus the nervous susceptibility is very much augmented, the frequency and strength of the pulse are increased, and the animal heat is very much developed. These effects require a considerable dose of the stimulant. A smaller dose than is necessary to produce this effect, may serve to stimulate the lining membrane of the stomach to a more copious secretion of its digestive fluid, and the muscular fibres to a greater activity, so that the processes of chymification and chylification may be facilitated and accelerated, and the various secretions and excretions may be more readily affected. Should the dose, however, be carried too far, irritation or even actual inflammation may be excited in the part with which the stimulant comes in contact, and a state of fever may be induced, whereby many of the functions above enumerated, especially the secretions and excretions, may be very much retarded." 195.

Their action on the digestive organs is first considered.

"*Digestive Organs.*

"*Physiological State.*—Stimulants act in two ways on the digestive organs. 1st. Immediately on being taken they stimulate the stomach and intestines, the liver, pancreas, &c., by their local and direct action, as also the nervous centres, so closely connected by sympathy with the gastric organ. 2nd. When the stimulating principles have been carried into the torrent of the circulation, and penetrate with the blood into all the organic tissues, they exert their stimulating powers on the stomach, intestines, &c.

"The influence of these substances on the digestive organs is very manifest when they are taken internally; a sudden development of vitality takes place in the epigastric centre. A feeling of heat, referred by the individual to the stomach, indicates the species of effect which is then produced in this organ; the muscular coat of this viscus is made to contract, so that its cavity becomes diminished. Hence the functions of the stomach are aroused; if it be empty, a craving for food is observed to take place; if the individual be just after eating, the powers of the stomach are called forth, so that the food is elaborated with unusual rapidity. The same effects are produced in the intestines. The secretions of the liver are also promoted and increased by substances of this class."

"*Pathological State.*—When the parietes of the stomach and intestines are attenuated and atrophied, the effects of stimulants are, in general, less striking. Still, even in this case, they excite the languid appetite, facilitate and accelerate chylification, prevent the unpleasant symptoms which sometimes accompany digestion in such cases, and remove constipation. Where the stomach and intestines are hypertrophied, the power of stimulants is very great: they are then observed to render the appetite voracious beyond measure." 196.

Next comes their action on the circulating organs.

"Most stimulants exercise a powerful action on the heart. After their employment the blood contained in the coronary arteries stimulates its tissue and accelerates its action, the contractions of the organ become more frequent, and are performed with unusual energy, impressing more force on the column of blood which passes through the arteries. These canals also appear to participate in the effect of the stimulant; they yield a more tense and firm stroke under the finger. The increased frequency of the pulse under the influence of stimulants is a matter acknowledged on all hands." 199.

Their action on the organs of respiration, urination, and on the nervous system, are now fully noted, and lastly, their therapeutical employment, is fully discussed.

In addition to general therapeutics, this little work contains a very convenient table of the different poisons arranged under Dr. Christison's three great heads, of *Irritants, Narcotics,* and *Narcotico-acrids,* with their names, symptoms, and treatment.

Many useful hints also are to be found in the remarks appended on the "*Rules to be observed in Prescribing.*" Dr. S. shews the objects in view in prescribing—the objects in view in the combination of medicines, and points out the faults which frequently occur. He describes the different forms of medicines, the quantities they should contain, and concludes his work with a very large collection of prescriptions, arranged appropriately, according to their effects.

We have been much gratified with this little work. Our readers, we are sure, will derive much pleasure, and much useful information from its perusal.

### II. Elements of Materia Medica and Pharmacy.

Before reviewing the contents of the work before us we should be glad to be quite clear to whom to refer it; and, we must confess we are not a little perplexed on this point. We turn to the title-page and read,—Elements of Materia Medica and Pharmacy, by Dr. O'Brien Bellingham, edited by Dr. Mitchell. The problem appears solved at once. The work is Dr. Bellingham's and Dr. Mitchell has only the merit of editing it. But let us now turn to the Preface—the editor's preface we presume, and how read we? We now find that the present work is to be considered as the "*substance of Dr. Bellingham's lectures*" on materia medica. This materially alters our views of Dr. Mitchell's editorship; and we are now to consider that the bare classifications of Dr. Bellingham, delivered in his lectures, have been arranged and digested into the present volume, under the superintendence of Dr. Mitchell. The whole compilation, therefore, of the work is due to Dr. Mitchell, and Dr. Bellingham has only the merit of having suggested the best "*method of classifying*, and the *best method of treating* the *several subjects*."

The preface is short, and we shall transcribe it, in order that our readers may deduce, (if they can) the plan of Dr. Mitchell's work.

"*Preface.*—The Editor of these Elements of Materia Medica, and Pharmacy, (having been engaged for many years in teaching the different branches of medicine,) has frequently experienced the want of a work upon these subjects, which he could safely recommend to the student; not that there was any remarkable scarcity either of elaborate treatises, or of manuals of the materia medica; but a work was wanting, which, in filling up the deficiencies of the majority of compendiums, and manuals, should include everything of importance contained in the more elaborate treatises; should supply the omissions which occur in the latter; and without overloading the pages with unnecessary details upon the one hand, or distracting the attention by speculative, or theoretical opinions, upon the other; should contain everything really essential upon these subjects.

"This deficiency, the Editor has endeavoured to supply, having been familiar for several years, with the arrangement followed by Dr. Bellingham in his Lectures upon the Materia Medica; and with the manner in which the several subjects were treated; the former being superior to any classification with which he is acquainted; and the latter presenting some novelty, and appearing to possess some superiority over those generally adopted; he has undertaken the editing of the present work, (which contains the substance of these Lectures,) with the hope that in so doing, he is supplying a deficiency in medical literature, and in the expectation that he is conferring a substantial benefit upon the medical student."

Our readers will observe that the editor has no small task to perform. To produce a work, filling up all the deficiencies of former works, and yet containing no unnecessary details, and yet everything essential, requires, to say the least, considerable judgment. We have looked through the present work with great care, and we cannot compliment Dr. Mitchell in the attainment of these objects; we cannot think that the present work, unless considerably revised, will confer that substantial benefit on the medical student to which Dr. Mitchell aspires. But to enter a little into detail. Our readers (we hope) have found out from the preface the plan of the work. We can assure them we have been at not a little trouble to do so. We had anticipated *some* sort of arrangement of the materia medica to start with, but we have been disappointed. In the last chapter, indeed, which occupies eighty-five pages, Dr. Mitchell introduces enough *classifications*: there is list upon list of the materia medica; but before we get to this chapter, we cannot conceive what arrangement our editor is following. The first two chapters contain the physico-chymical histories of the different *elementary* bodies, the compounds of which are found as officinal preparations, and we presume Dr. Mitchell only intends to give us their physico-chemical history without any reference to their pharmaceutical uses. For not a preparation of these elements is given. We are not finding fault with *these* details; but we would know in what they differ from similar details in works professedly written on chemistry.

Chapters 3, 4, 5, and 6, contain the chemical history of *compound substances* of which officinal preparations may be made—*e. g.* metallic oxides, under which are included the acids and alkalies; also the general properties of salts; and the chemical history of the proximate principles of vegetables. These last occupy the two last chapters, and are arranged according to the classification of Dr. Thompson, viz. into 1, neutral vegetable principles—2, intermediate bodies—3, vegetable acids—4, vegetable alkalies.

These several compounds are treated with reference to their chemistry alone; we cannot see what particular object Dr. Mitchell has in treating these subjects without any reference whatever to their applications in pharmacy.

Chapter 7, is occupied with the general nature of pharmaceutical operations; and the several operations of pharmacy, viz. division, pulverization, trituration, levigation, sifting, eleutriation, filtration and expression. are most minutely described.

Chapter 8 is a chapter of some length on pharmaceutical preparations, including a general view of the methods of their formation, with a list of all the different *tinctures, powders,* &c. contained in the different Pharmacopœias of Great Britain, under each head respectively. These lists, we need scarcely inform our readers, are found in the Pharmacopœias themselves, and we cannot help including them among several other, what we think, "*unnecessary details.*" The remarks above-mentioned, however, are premised by some observations on the methods of collecting, drying and preserving indigenous plants.

We are introduced in Chapter 9, to the modus operandi of medicines; and Chapters 10 and 11 contain the circumstances, such as age, sex, and temperament and habit, which modify the action of medicines, and the modes of their administration, with a few rules for prescriptions. We have alluded to Chapter 12, on Classification, which concludes this Part.

The above, we are aware, is a mere summary of the contents of the present work, and we intended it to be so.

We wished our readers, by reading *our* little preface, to be saved the trouble of wading through the whole work to see its general scope. We have now to shew, how Dr. Mitchell has fulfilled his promise of "filling up deficiencies of smaller works," and, "introducing all that is necessary, with no unnecessary details" into his own. We would ask any of our young friends studying materia medica—for what do you refer to a work on materia medica? Would you not wish among other things to know something about the pharmaceutical preparations. You may look in vain for them in the present work. We will transcribe the history of iron.

"CLASS II.—METALS THE OXIDES OF WHICH ARE NEITHER ALKALIES NOR EARTHS.

ORDER 1.—Metals which decompose water at a red heat.

FERRUM—IRON.

*(Derived from ferire, to strike.)*

"*History.*—Iron was known in the early ages of the world; its antiquity, however, is not so great as that of copper, silver, or gold; it is a constituent of the animal and vegetable, as well as of the mineral kingdom, and is very abundantly diffused in nature. Iron rarely occurs in its pure metallic form in the earth, most commonly in combination with oxygen or sulphur, or with oxygen and an acid.

"The ores from which iron is extracted are (in England) clay iron ore, a carbonate of the protoxide, which occurs chiefly in the coal formation; and (in Sweden and India) red hœmatite, and brown hœmatite, both of which are peroxides, and magnetic iron ore, or the black oxide, which occur in primary rocks, and afford the finest kind of iron.

"*Preparation.*—The clay iron ore is first roasted, by which carbonic acid is driven off; it is then exposed to an intense heat in a blast furnace, mixed with charcoal, or coke and limestone; the carbonaceous matter deprives the ore of oxygen, and the limestone acts as a flux, combines with the earthy matters, (alumina and silica) into a fluid compound, which remains upon the surface, and afterwards constitutes the slag; while the fused metal accumulates at the bottom, and is run into moulds, where it forms what is called cast-iron, or pig-iron, in which state it contains several impurities; it afterwards undergoes the several processes of refining, puddling, and welding, before it is converted into malleable iron.

"*Properties.*—Iron is a hard metal, of a whitish grey colour when pure, and susceptible of a high polish; its taste is styptic; its texture fibrous; it is less malleable than several other metals, but possesses considerable ductility, and in tenacity it exceeds every other metal; its specific gravity is 7.7; it is attracted by the magnet, and is capable of becoming magnetic—a property possessed by but one other metal, nickel; when heated to redness it becomes soft, and in this state is capable of being welded; it requires a very intense heat for fusion, and has never been volatilized. Iron has a strong affinity for oxygen, and attracts it from the air, when moisture is present, or when heated in the open air; it burns in oxygen gas with vivid scintillations; it is oxidized by dilute sulphuric acid, at the expense of the water, with evolution of hydrogen gas; nitric acid oxidizes it, at the expense of a part of the acid, Iron decomposes water at common temperatures, but slowly; at a red heat the decomposition is rapid, hydrogen gas being evolved. Iron unites with oxygen in two definite proportions only, forming a protoxide and a peroxide. The black or magnetic oxide, which was formerly supposed to be the protoxide, is a mixture of protoxide and peroxide.

"The chemical equivalent of iron is 28.

"PROTOXIDE OF IRON.

"*Preparation and Properties.*—Protoxide of iron is always formed when iron is acted on by dilute sulphuric acid; it is the base of protosulphate of iron, or the green vitriol of commerce, and of the native carbonate of iron. If we add caustic potassa to a solution of protosulphate of iron, a white precipitate falls, which is the hydrate of the protoxide; it speedily, however, absorbs oxygen, changes colour, becomes green, and finally brown red; hence the protoxide of iron cannot be procured in an insulated state.

Its chemical equivalent is 36: it is composed of one equivalent iron 28, and one equivalent oxygen 8.

" *Characteristics.*—1. The salts of the protoxide of iron have a green or bluish green colour, an astringent taste, and redden litmus paper; they are usually soluble in water, and all are soluble in dilute hydrochloric acid.

" 2. Protoxide of iron is thrown down from its salts, as a white hydrate, which speedily changes to green and brown red, by caustic potash or ammonia.

" 3. Carbonate and bicarbonate of potash, and carbonate of ammonia, precipitate it, as a white carbonate of the protoxide, which by exposure to the air becomes red.

" 4. Phosphate of soda throws it down, as a white phosphate, which becomes green on exposure to the air.

" 5. Ferrocyanuret of potassium (yellow prussiate of potash), throws down a white precipitate of ferrocyanuret of the protoxide, which becomes blue by exposure to the air.

" 6. Ferrosesquicyanuret of potassium (red prussiate of potash), throws down a deep blue precipitate of Prussian blue.

" 7. Hydrosulphuret of ammonia produces a black precipitate of protosulphuret of iron.

" 8. Hydrosulphuric acid (sulphuretted hydrogen,) or infusion of galls, produce no precipitate.

" 9. The double chloride of sodium and gold produces a purple precipitate.

" PEROXIDE OF IRON.

" *Preparation and Properties.*—Peroxide, or red oxide of iron, occurs in nature, under the forms of red and brown hematite. The rust of iron and protocarbonate, by exposure to the air, absorb oxygen, and are converted into peroxide of iron. It may be prepared artificially by exposing sulphate of iron to a high temperature, by which the water and acid are driven off, and the protoxide is converted into peroxide; or by dissolving iron in nitrohydrochloric acid, and adding an alkali; when a bulky brown precipitate of the hydrated peroxide of iron falls down, which when dried assumes a deeper colour. The peroxide of iron combines with acids to form salts, but is a weak base, as its salts are easily decomposed.

" Its chemical equivalent is 80; it is composed of two equivalents iron 56, and three equivalents oxygen 24.

" By some chemists it is called the sesquioxide of iron.

" *Characteristics.*—1. The salts of the peroxide of iron have a red or brown colour, an astringent taste, and an acid re-action upon litmus paper; those which are insoluble in water dissolve in hydrochloric acid.

" 2. The peroxide of iron is precipitated as a bulky reddish brown hydrate, by caustic potash, soda or ammonia, by carbonate, and bicarbonate of potash, and carbonate of ammonia.

" 3. Ferrocyanuret of potassium throws down Prussian blue; but the ferrosesquicyanuret produces no precipitate.

" 4. Gallic and tannic acids, and infusion of galls, produce a bluish black precipitate.

" 5. Sulphocyanic acid, and sulphocyanuret of potassium, produce a blood red precipitate; and meconic acid a wine red precipitate.

" 6. Hydrosulphuric acid (sulphuretted hydrogen) produces a milky precipitate of separated sulphur, and the peroxide is converted into the protoxide of iron." 51.

The "*deficiencies*" of other works are indeed filled up in this interminable description of iron; but we question if all that is "*necessary*" is introduced. The details here, it will be observed, include the physico-chemical properties of the metal; but we have to learn *from other works* the history of iron considered as an article of the materia medica.

As to the "unnecessary details" we are quite of Dr. Mitchell's opinion with reference to works in general. So much unnecessary detail is often found, that it is difficult at times to detect the real substance-matter. But we cannot help feeling that the present volume is not quite free from the same criticism.

For example: before the individual history of each metal above alluded to, we have the following remarks on the general properties of metals.

"The metallic elementary bodies are much more numerous than the non-metallic; forty-two being known to chemists; only a small proportion of them, however, are possessed of medicinal virtue.

"The metals are distinguished by the following characters:—They are all opaque, good reflectors of light, and in general possess the metallic lustre; they are all very good conductors of heat and electricity; and when the compounds which they form with many simple substances are submitted to the action of galvanism, the metal appears always on the negative side; hence they are all *electro-positive substances.* Many of the metals are remarkable for great sp. gr.; gold and platinum are the densest bodies in nature; the former being 19 the latter nearly 21 times heavier than an equal bulk of water: a few metals however, are very light, potassium and sodium float upon water. Some metals possess the property of malleability, that is, admit of being beaten, or rolled out into thin leaves, depending upon a high degree of tenacity in the metal, with a certain degree of softness; gold is the most malleable of the metals; silver, copper, tin, and lead rank next in malleability. Most of the malleable metals are also ductile, that is, admit of being drawn out into wire; the ductility of a metal, however, is not in all cases proportionate to its malleability; iron is a very ductile metal, but not very malleable. The tenacity of a metal is determined by the weight which a wire of a given thickness will support, without breaking: iron possesses this property in the highest, and lead in the least degree. The majority of the metals have neither taste nor smell; a few, however, when rubbed, or when their temperature is very slightly elevated, have a peculiar odour and taste, as copper, iron, and tin. Metals are usually hard; lead, however, is soft, and potassium yields to the pressure of the finger; a few, are brittle, as bismuth, antimony, and arsenic.

"All the metals, with the exception of mercury, are solid at ordinary temperatures; they may all, however, be fused by heat, but the temperature at which they liquefy is very different, thus potassium fuses at 136° of Fahrenheit's thermometer, tin at 442°, bismuth at 497°, lead at 612°, zinc at 773°, silver at 1873°, copper at 1996°, gold at 2016°, iron requires the highest heat of a smith's forge before it fuses; and an alloy, composed of two parts by weight of bismuth, one of lead, and one of tin, fuses when heated a little above 200°, and consequently melts in boiling water: it is called the fusible metal from this circumstance. Metals differ also from one another in volatility; the majority are highly fixed substances, while some, as arsenic, zinc, and mercury are readily volatized. Metals, like other solids, expand and contract when exposed to changes of temperature; but the degree of expansibility varies, each metal possessing a rate of expansion peculiar to itself: thus lead expands in volume one 350th, and iron one 800th, in other words 350 cubic inches of lead become 351, and 800 of iron

801. In some common operations the expansion and contraction of a metal are taken advantage of, as in hooping casks, and applying the tire or iron band to carriage-wheels, the iron is made, in the first instance, too small to fit, it is then heated until it expands sufficiently, and applied; when it is suddenly cooled by throwing water upon it: the contraction produced by which is so great, that the parts are bound together with exceeding firmness. Metals appear to be more susceptible of changes of bulk, by variations in temperature than other solids; hence their expansion by heat and contraction by cold afford the means of measuring degrees of temperature. The instrument used for this purpose is termed a thermometer, (derived from θερμος heat, and μετρον measure.) Mercury is the metal usually employed for thermometers; but for measuring very low temperatures, alcohol is used, as it cannot be frozen; and for temperatures above that at which mercury boils, an instrument termed a pyrometer (derived from πυρ fire, and μετρον measure) is employed.*

"Metals differ from one another in colour; the majority have a grey or bluish white tinge: gold, however, is yellow, copper red, bismuth has a reddish tinge, and silver is white. Their texture also varies; iron is fibrous, zinc and antimony are lamellated, and many of them are capable of crystallizing; this is best effected by fusion and slow cooling. The most usual forms of the crystals of the metals are the cube and octahedron.

"Metals combine, or may be made to combine with one another, giving rise to the important class of compounds denominated alloys, (in those cases in which mercury enters as an ingredient, the compounds are termed amalgams.) The alloys in most common use are brass, and pinchbeck, combination of zinc and copper; bronze and bell-metal, combination of tin and copper; tin plate, an alloy of tin and iron; pewter, a combination of lead and tin. The standard gold and silver of commerce are alloys of gold or silver, with a little copper. Metals unite also with several other simple substances; they all combine with oxygen, sulphur, and chlorine; many of them also combine with iodine, phosphorus, cyanogen, &c. &c.

"A few of the metals, as gold, silver, copper, bismuth, and mercury, occur in nature in the pure metallic form, or alloyed with other metals; the majority, however, are only found in combination with other substances, particularly sulphur and oxygen, in which state they are said to be *mineralized*; and the native compounds of the metals are termed their *ores*." 34.

We must confess we cannot but think that these remarks are irrelevant

---

"* Fahrenheit's Thermometer is the one in common use in this country. Its scale commences with the temperature produced by mixing snow with salt—that is 32 degrees below the freezing point of water, (which at the time was supposed to be the lowest degree of artificial cold.) The space between this, zero or 0°, and the boiling point of water, is divided into 212 degrees; consequently in Fahrenheit's scale water freezes at 32°, and boils at 212°. In Reaumur's Thermometer, which is used in Germany, the freezing point of water is zero or 0°, and the boiling point 80°; and in the Centigrade Thermometer, which is generally employed in France, the freezing point of water is zero or 0°, and its boiling point 100°. Mercurial thermometers may be employed to indicate degrees of temperature between 20 or 30 below zero, and 580° of Fahrenheit; above this point the mercury boils in the bulb of a thermometer hermetically sealed, (not being subject to atmospheric pressure.) An alcohol thermometer cannot be employed to indicate a temperature above 180°; if subjected to a higher temperature it will burst, owing to the conversion into vapour of the alcohol; but, as already observed, it may be employed for a much lower temperature than a mercurial thermometer."

in a work on materia medica. As to the note on the thermometer, it would have been out of place in a work on chemistry.

We turn to Chap. IV. Dr. Mitchell has been describing the general properties of salts. He has defined a salt—has mentioned some of the properties of salts: effervescence, for example, deliquescence, &c. He has shown the difference between neutral salts, subsalts, and supersalts, and, after telling us that ic-acids produce ate-salts and ous-acids ite-salts,* he finishes his account of salts with the two following paragraphs.

"The combinations of chlorine, iodine, and bromine, with metals, are distinguished by the termination *ide:* thus we have a chloride, iodide, and bromide of potassium. When the metal forms more than one compound with any of these bodies, they are distinguished in the same manner as the salts of the oxides, by the prefix *proto, bi, per di:* thus we have a protochloride and a bichloride of mercury, a dichloride of copper, and a protiodide, sesquiodide, and biniodide of mercury.

"The combinations of sulphur, phosphorus, &c. with metals, are distinguished by the termination *uret:* and when they combine in more than one proportion with a metal, they are distinguished by the prefix *proto, bi, ter, sesqui,* as protosulphuret and bisulphuret of mercury, sesquisulphuret of antimony, &c." 86.

We are perfectly at a loss to know what these remarks have to do with the subject of salts, much more what is the object of their introduction in a work on the materia medica.

We have alluded to pages of (what we think) "unnecessary details," in the lists of the pharmaceutical preparations. The tinctures of the Pharmacopœias arranged alphabetically, occupy nearly a page. The ointments more than half a page; the decoctions about half a page; the others, syrups, infusions, mixtures, &c. &c. more or less space.

We did not gain any information from these lists; and we could not help thinking that pages full of well-digested remarks on the peculiarities in the modes of preparation of individual tinctures, extracts, &c. would have well supplied their place.

But we must not quit this work without some notice of the style of the author. It is peculiar—we cannot propose it for imitation. We cannot compliment Dr. Mitchell either on its perspicuity or its accuracy. Our readers must have formed a tolerable idea of it from the perusal of the preface. We shall introduce them to a few other singular passages. "Metals by combining with oxygen (observes Dr. Mitchell, Chap. 3,) give origin either to oxides, acids, or alkalies. The alkalies, with the exception of ammonia, the earths, and the alkaline earths, being combinations of a metallic base with oxygen, are now considered to be metallic oxides." Is ammonia to be considered a combination of a metallic base with oxygen? or are the earths and the alkaline earths to be considered as alkalies?

This passage is followed by another, which contains some gross libels on both the Greek and Latin tongues.

"The same metal is susceptible in many cases of combining with different proportions of oxygen to form oxides; and these are distinguished from one

---

* All these remarks are appropriate enough in a work on chemistry; but we question whether we should have introduced them in a work on materia medica.

another by a derivation from the Greek being prefixed, as *Pro, Bi,* or *Deu, Tri, Ter,* or *Per;* the oxide which contains the minimum of Oxygen, is termed a *Protoxide,* and that which contains the largest proportion, a *Peroxide.*" 75.

All these prefixes are from the Greek! Our editor would flounder for some time in a Greek Lexicon even for pro, deu, or tri, meaning 1st, 2nd, and 3rd—longer still for bi or ter—and we are surprised that he should quote *prot*oxide as an example of the application of the prefix *pro.* This passage is followed by another explaining the meaning of the term *sesqui.*

"The term *Sesqui* is also used to designate an oxide, which has little or no tendency to unite with acids, and which contains *less** than one atom of oxygen to one atom of the metal; thus, when the proportion of oxygen in three oxides of the same metal, is in the relation of 1, 1½, and 2, the second is termed a *Sesquioxide.*" 75.

Had such an explanation occurred in an elementary work on chemistry, we should have been disposed to have considered it a blunder—we are only surprised to find such points as these dwelt on, in a work on materia medica.

"The metallic oxides," Dr. Mitchell continues, "are distinguished by the property of combining with acids to form salts;" we would ask, are distinguished from what? If this be their grand distinguishing property, then surely ammonia, morphia and brucia are metallic oxides.

We have before alluded to Chap. 4, as containing the general properties of salts. We now subjoin Dr. Mitchell's definition of a salt.

"A salt is a definite compound of an acid and an alkaline, or salifiable base, in which the properties of each of these bodies are neutralized; and the resulting compound possesses neither the character of an acid, nor of an alkali; but chemical and other qualities, distinct from either. Several of those substances, which are commonly termed salts, are not composed of an acid and an alkaline, or salifiable base, as chloride of sodium, (a compound of chlorine with a metal) and are not consequently included in this definition. These bodies, however, are now grouped together in a separate division of the salts, under the name, haloidal salts." 82.

A *salt* is a definite compound of an acid and alkali; but several of these substances commonly called salts are not salts, and yet they are grouped together in a separate division of *salts,* and called haloidal salts. How is a student to know what a salt is?

On the same subject of salts Dr. Mitchell observes. "The same quantity of water may hold several salts in solution, provided they have no tendency to decompose each other; and, on evaporation of the water (provided they have no tendency to combine together), the salts will crystallize separately. In many cases, however, they mutually decompose each other, and two new salts are formed." The formation of *two* new salts by the mutual decomposition of *several* is not common.

Again Dr. Mitchell observes, concerning the vegetable acids:—

"*History.*—The vegetable acids form a very numerous division of the proximate principles; as many as 116 have been examined by chemists; they are

---

* Dr. Mitchell prints *less* in italics.

named also organic acids, (which term includes the animal acids) to distinguish them from those of mineral origin. Some of these acids exist ready formed in vegetable substances, in combination with alkaloids; and others are formed artificially." 123.

We could not help smiling at the introduction of the parenthesis. Are animal acids then species of vegetable acids. Also, will not the appellation of vegetable acids sufficiently distinguish them from those of mineral origin?

Many other inaccuracies occur in various parts, such as considering cinchona and guaiacum to contain volatile principles, p. 153; considering *gutta* and *minimum* as identical, p. 219; and we are of opinion that a careful revision of the present part should precede the appearance of the second.

---

### III. A Dispensatory, &c.

Dr. Christison's Dispensatory is a commentary on the Pharmacopœias of Great Britain, comprising the natural history, description, chemistry, pharmacy, actions, uses, and doses of the articles of the materia medica.

The articles of the materia medica are arranged alphabetically. Before commencing their description, Dr. Christison presents us, in an Introduction, with some of the General Processes in Pharmacy.

His object is not a systematic treatise on the Preparation of Medicines, but "to describe a few articles of apparatus, which have been recently introduced into the art of chemical pharmacy, and without which the results aimed at by the colleges cannot, as appears to the author, be with such certainty, attained."

His observations, in fact, are running commentaries on the pharmaceutical instructions of the Pharmacopœias.

Under the head of *Acids* we have brought before us a convenient apparatus, Liebig's Refrigeratory, for condensing all vapors which do not act on cork. It is very simple, and in the above-mentioned cases of distillation is to supersede Wolfe's apparatus.

To shew the style of the remarks in this Introduction we subjoin his section on the Alkaloids.

"The vegetable alkaloids may be obtained by a variety of processes; which invariably however comprehend decomposition of the alkaloidal salt in the crude drug, either by the superior affinity of an alkali, earth, or alkaline carbonate, or by double decomposition with some compound salt, whose base forms an insoluble salt with the acid in the drug. Active neutral principles, such as narcotin from opium, piperin from white pepper, picrotoxin from cocculus-indicus, and elaterin from elaterium, may be obtained through the agency of such simple solvents as water, rectified-spirit, and sulphuric ether, used singly or successively.

"In the formula for preparing veratria recommended by the Edinburgh College the process of exhaustion by percolation is mentioned for the first time in the Pharmacopœias. Some experience is required to apply this process in all cases with success. But, when well performed, it is greatly superior in general to every other mode of extracting the active matters of vegetable drugs, where the liquid used is spirituous or etherial; and it is often not less advantageous in the instance of water, as well as acidulous fluids. The precautions for applying it successfully will be considered under the head of Tinctures." iv.

The principle of obtaining the alkaloids is here *very* generally exhibited. The particular process for each alkaloid is to be met with under its appropriate head. The process of per-colation mentioned above, is not employed in the London Pharmacopœia, but the following general directions are given for it by the Edinburgh College.

TINCTURES.

"A much superior method however, has been lately introduced, which answers well for most tinctures, namely the method of displacement by percolation. According to this process, the solid materials, usually in coarse or moderately fine powder, are moistened sufficiently with a solvent to form the thick pulp ; in twelve hours, or frequently without any delay, the mass is put into a cylinder of glass, porcelain, or tinned-iron, open at both ends, but obstructed at the lower end by a piece of calico or linen, tied tightly over it as a filter ; and the pulp being packed by pressure, varying as to degree with various articles, the remainder of the solvent is poured into the upper part of the cylinder, and allowed gradually to percolate. In order to obtain the portion of the fluid which is kept in the residuum, an additional quantity of the solvent is poured into the cylinder until the tincture which has passed through equals in amount the spirit originally prescribed; and the spirit employed for this purpose is then recovered for the most part by pouring over the residuum as much water as there is of spirit retained in it, which may be easily known by an obvious calculation in each case. The method by percolation, where applicable, will be found much more convenient and expeditious than the mode hitherto commonly followed ; and it exhausts the solid matters in general much more completely. As considerable practice however is required for managing the details in different cases, more especially in regard to the degree of minuteness of division of the solids, and the degree of firmness with which they are to be packed in the cylinder, we have thought it right to direct that the method by maceration may be followed as an alternative. But the method by percolation is now preferred by all who have made sufficient trial of it to apply it correctly." xxi.

Dr. Christison comments on this process, and considers it the best, and to the experienced it undoubtedly is so, and to those druggists and others who purpose trying it, the above observations with the accompanying simple apparatus, which is figured, will be found very useful. It is troublesome, however, and the London method of maceration for fourteen days, and straining, although it may not, in all cases, quite exhaust the substance of its soluble ingredients, yet certainly makes very good tinctures.

We are a little disappointed with the criticisms on the vegetabilium preparatio. Neither the Edinburgh nor the Dublin College give any general directions for the collection of indigenous plants. The London gives the following, which we believe to be founded on strictly physiological principles.

"GENERAL DIRECTIONS, *Lond.* Vegetables are to be gathered in dry weather, when not wet with showers, or dew. They ought to be collected annually, and thrown away when kept longer than a year.

"*Roots* are dug up for the most part before the appearance of the stems or leaves.

"*Barks* should be collected at the season when they are most easily detached from the wood.

"*Leaves* should be gathered after the flowers are expanded, and before the seeds ripen.

"*Flowers* should be plucked when fresh blown.

"*Seeds* are to be collected when ripe, and preserved in their own pericarps." xxi.

Dr. Christison observes:—

"The collection of vegetable simples is an occupation that requires more skill and knowledge than are usually possessed by those who follow it." "The principles now current have been succinctly stated by the London College in its directions printed at the head of this article. But the doctrines there espoused are subject to numerous exceptions; and other modifying circumstances connected with vegetation might be mentioned, such as season, climate, weather, site, soil, exposure, cultivation, which have not been adverted to at all,—probably because the College felt that their respective influences have not been determined with such accuracy, as to admit of being expressed in general rules." xxvi.

Dr. Christison adds;—

"It is surprising how little attention this whole subject,—interesting alike in a practical point of view, and in relation to the physiology of plants,—has hitherto received from scientific men. A few years ago I commenced an experimental inquiry on some departments of it, which other occupations compelled me to suspend. But I had gone quite far enough to be convinced, that the doctrines commonly held, as to the influence of various circumstances in vegetation on the activity of medicinal plants, are often erroneous, and generally subject to important exceptions; that the practical rules founded on those doctrines are sometimes faulty; and that the pharmaceutic art stands much in need of a new and thorough investigation of this imperfectly trodden field." xxvi.

We merely observe that these remarks add little to our previous information on this subject.

We proceed now to the review of the special articles on individual drugs. Each is complete in itself. We select two, as specimens.

"CONIUM, *E. D.* CONII FOLIA, *L.* *Leaves of Conium maculatum, L. W. DC. Spr. Hemlock.*

"Tests, *Edin.* The powder triturated with Aqua potassæ, exhales a powerful odour of conia.

"CONII FRUCTUS, *L.* *Fruit of Conium maculatum, &c.*

"Extractum Conii, E. L. Succus Spissatus Conii, D.

"Process, *Edin.* Take of

Conium, any convenient quantity; beat it into a uniform pulp in a marble mortar, express the juice and filter it. Let this juice be evaporated to the consistence of firm extract either in a vacuum with the aid of heat, or spontaneously in shallow vessels exposed to a strong current of air freed of dust by gauze screens.

"This extract is of good quality only when a very strong odour of conia is disengaged by degrees on its being carefully triturated with aqua potassæ.

"Process, *Lond. Dub.* To be made from fresh leaves, like extract of Monkshood.

"Tinctura Conii.

"Process, *Edin.* Take of

Fresh conium-leaves, twelve ounces;

Tincture of cardamom, half a pint;

Rectified spirit, a pint and a half.
Bruise the leaves, express the juice strongly; bruise the residuum, pack it firmly in a percolator: transmit first the tincture of cardamom, and then the rectified spirit, allowing the spirituous liquors to mix with the expressed juice as they pass through; add gently water enough to the percolator for pushing through the spirit left in the residuum. Filter the liquor after agitation.
" PROCESS, *Lond. Dub.* Take of
Dried conium leaves, five ounces (two, D.);
Cardamom, bruised, an ounce;
Proof-spirit, two pints (a pound by measure, D.)
Macerate for fourteen days (seven D.) and strain.

"UNGUENTUM CONII, D.

" PROCESS, *Dub.* Take of
Fresh conium leaves, and
Prepared lard, of each two pounds.
Boil the leaves in the lard till they shrivel; strain through linen.

"CATAPLASMA CONII, D.

" PROCESS, *Dub.* Take of
Dried conium leaves, an ounce;
Water a pound and a half by measure.
Boil down to a pound, and add to the strained liquor enough of the same powder to make a poultice.
" FOR. NAMES.—*Fren.* Grande cigue.—*Ital.* Cicuta maggiore.—*Span.* Cicuta; Ceguda.—*Port.* Cigude.—*Ger.* Gefleckte Schierling.—*Dut.* Dollekervel; Scheerling.—*Swed.* Spräklig odört.—*Dan.* Skarntyde.—*Russ.* Boligolov piatnistoi.
" FIGURES of Conium maculatum in Hayne, i. 31.—Nees von E. 282.—Steph. and Ch. i. 13." 359.

These useful and necessary particulars introduce the article. Conium is treated under the five following heads. 1. Its general history and introduction into medicine. 2. Its natural history. 3. Its chemical history. 4. Its adulterations. 5. Its actions and uses.

The general opinion certainly is that conium is identical with the Κωνειον of the Greeks. Dr. Christison, however, discredits this. He considers that the description by Dioscorides, differs materially from the characters of the conium maculatum of modern botanists, and that the accounts given by Nicander and Plato of its action as a poison do not tally well with what is now known of its effects, and the ancient name cannot be traced, like others, in the language of the modern Greeks, who call the true plant Βρομοχορτον.

" *Natural History.*—The plant is a biennial, belonging to the natural family *Umbelliferæ* and to Linnæus's class and order *Pentandria Digynia.* It is met with abundantly in this country, as well as in most parts of the Continent, along hedges and roadsides, and on exposed banks of rubbish. In its first season it consists of a long slender root and a few small root-leaves spread flat upon the ground. Early in its second summer it has a solid, white, ligneous and amylaceous root towards two feet or upwards in length; and it afterwards pushes up a flowering stem commonly between two and four feet, sometimes even six feet, in height, hollow, jointed, and speckled with numerous dark purple spots. Its leaf-stalks are similarly speckled. The fruit which ripens in August and September is plano-convex, with five undulated ridges on its convexity. The whole plant is smooth and destitute of hairs or down. It emits, especially in sunshine, a strong peculiar odour like that of mice,—which by many authors has been incorrectly likened to that of cat's urine. It bears a general resemblance to various umbelliferous plants, and has been confounded by the unskilful with *Æthusa cynapium, Œnanthe crocata, Cicuta virosa, Myrrhis odorata, Myrrhis temulenta,* and some other plants. In its second summer it may be easily distinguished by its purple-spotted stem and leaf-stalks from every species except the *Myrrhis temulenta,* a very common inhabitant of roadsides; and that species is at once known from it by every part of the plant being hairy. In its first season, and also in its second until the month of May, when it first begins to push up its flowering stem, the leaf-stalks are seldom spotted; in which condition its best character, for those not familiar with its appearance, is the strong peculiar odour exhaled when the leaves are bruised with solution of potash." 360.

Dr. Christison now adverts to the period of gathering the leaves and fruit. He expresses doubts as to the best period. According to the authority of Dr. Fothergill, "the leaves are in the most active state, and ought therefore to be gathered for medical use, when the plant is in full flower, or *even a little later, when the flower is passing away and the fruit is forming.*" "This rule, I suspect, (says Dr. Christison) is far from being so certain as many think. And from such experiments as I have made on its poisonous properties, there would appear to be no great difference at any season; for even in November and March of *its first year* its activity is very great."

We are far from depreciating any *observations* accurately made, however averse to deductions from physiological principles; but we cannot help thinking, that if the directions of the London Pharmacopœia, with respect to the gathering of leaves above-mentioned, were adopted in reference to conium, we should get them in full vigor. The mere floral envelopes in umbelliferæ are very insignificant, and will take but little nourishment for their formation. But the grand object of the plant in flowering, as far as the present point is concerned, is to concentrate its principles in the seed, the miniature representative of the species. The juices of the whole plant, leaves among the rest, are consequently employed in this object—hence they should be gathered, as the College direct, before the seeds ripen. Dr. Christison says, its activity is very great even in November and March of its *first year.* This is quite true, for the plant is a biennial; the leaves are then performing their office of assimilation, they contain the secretions of the plant, and as no seed is formed that year, they retain them till they die.

*Chemical History* is very complete. The two leading principles are *Conia* and a peculiar *Aromatic oil.* The volatile oil, obtained in the usual way by the distillation of the leaves with water, is remarkable, inasmuch as it is not poisonous. "In this respect (says Dr. Christison) hemlock presents one of the most distinguishing characters of the family to which it belongs." We must confess however, in examining this oil, we have always had a suspicion that it was a modification of conia, effected by distillation; for if there be an aromatic oil in the leaves, there will be (probably at least) a volatile oil in the fruit; and yet it is well known that the Conium Cremocarps have no vittæ for its reception.

"Conia is at first colourless, but is very prone to become brown by oxidation. It has an intense, peculiar, suffocating odour, and an extremely acrid, benumbing taste. Exposed to the air, it gradually becomes brown, disengages ammonia, deposits a resinous matter, and loses much of its activity. It boils at 370° and may be distilled in close vessels without alteration. It distils over with water at 212° like the volatile oils. It is sparingly soluble in water; but water unites with it in the proportion of about a fourth part to form a hydrate. It is very soluble in alcohol and ether, in the fixed and volatile oils, and also in weak acids, which it neutralizes. Its salts have not as yet been crystallized. It exists in the greatest abundance in the full-grown, green fruit; the ripe fruit contains less; the leaves still less; and the root very little. Eight pounds of green fruit will yield half an ounce of hydrated conia; but they contain much more, for a large proportion is obviously decomposed in the process.——Some have imagined that conia does not exist in the plant and is not a pure alkaloid, but is formed by the action of potash and owes its alkalinity to ammonia (Deschamps). This however is a mistake. For chlorine, which disengages azote from a weak solution of ammonia, has no such effect on solution of conia: and, like solutions of other vegetable alkaloids, the natural hemlock-juice gives with infusion of galls a precipitate, from which conia may be obtained by the ordinary processes for other alkaloids in the like circumstances (Boutron and Henri)." 362.

Two methods are given for obtaining it—one is extremely simple. "Cautiously distill from a muriate of lime bath a mixture of strong solution of potash with the alcoholic extract of the unripe fruit. The alkaloid passes over with the water, and floats upon it like an oil."

Dr. Christison shews the precautions necessary in preparing the ordinary *Extractum Conii*, the expressed juice inspissated by *heat*; the superiority of Mr. Barry's process of evaporation in vacuo; and the super-superiority of the process of the Messrs. Smith, who concentrate the expressed juice to a thin syrup in vacuo, and then finish the evaporation spontaneously. He also strongly recommends the tincture of conium to be made from the recent leaves, "by expressing the juice, transmitting rectified spirit through the residuum, mixing the watery and spirituous fluids, and filtering the product." This *must* be an improvement on the dried leaves.

After alluding to the *Adulterations*, we are brought to the Actions and Uses.

"*Actions and Uses.*—The actions of hemlock have been long misunderstood. It has been known immemorially as a narcotic poison of great virulence; and it was supposed to excite convulsions and fatal coma, to render the blood fluid, and to exhaust the irritability of the heart. I have endeavoured on the contrary to show, that it leaves the heart's action unimpaired, and does not prevent the blood from coagulating, any more than other causes of death by asphyxia,—that it does not excite convulsive spasms or bring on insensibility,—but that it exhausts the nervous energy of the spinal chord and voluntary muscles, occasioning merely convulsive tremors and slight twitches, and eventually general paralysis of the muscles and consequent stoppage of the breathing (Trans. Roy. Soc. Edin. 1836). I was unable to detect its active principle in the blood; but Mr. Judd has since been more fortunate. Mr. Judd's observations on its poisonous properties do not exactly correspond with the view now given of them; and among other statements, he remarked that the contractility of the heart is powerfully affected. I apprehend, that the most essential differences between us arise from his not having appreciated precisely the phenomena, which are intricate and difficult to follow. The effects of hemlock in medicinal doses are very imperfectly known. Since the time when it was currently adopted as a remedy in consequence of the recommendations of Störck, it has been generally thought to be, when administered internally, an anodyne, hypnotic and calmative, as well as to possess important deobstruent properties, especially in malignant tumors and ulcerations. It has likewise been held to be a diuretic, to diminish the venereal appetite, and to lessen the secretion of milk. Hence it has been employed in scirrhus, in cancerous and other malignant ulcers, in strumous sores and enlargement of the glands, in pseudo-syphilitic ulcers, eruptions and nodes, in chronic enlargements of the liver and spleen, in chronic catarrh, asthma and hooping-cough, and in neuralgia of all kinds. But the deobstruent virtues of hemlock are now almost universally discredited; its diuretic action is too unimportant to be available in practice; and little faith can be attached to what has been said by our predecessors of its influence in diseases of the nervous system, because the preparations they used were often inert, and the doses they administered too small to produce positive effects even though the preparations had been of good quality. The only careful investigations made since the conditions for obtaining good and uniform preparations were known are those of Mr. Judd (Med. Bot. Trans. 1839); who infers from experiments with medicinal doses upon cats and other animals, that a well-made extract causes great languor and drowsiness and often profound sleep for two or three hours, lessens muscular excitability, and reduces the circulation as well as the animal heat. He is therefore inclined to believe that hemlock really deserves the reputation it has enjoyed with many of being an efficient anodyne and hypnotic; he adds, that in some trials with it in patients affected with pectoral complaints he found it to have a salutary effect in allaying cough and promoting sleep; and he thinks it peculiarly applicable to the treatment of hypertrophy of the heart, phrenitis, and other affections attended with an excited or excitable state of the circulation. The few trials I have myself made with well-prepared extracts have not yielded such favourable results. I have occasionally found it to appearance useful as an anodyne and calmative in scirrhous uterus, but cannot say much for its virtues as such in neuralgic affections. On the whole it appears to me that the entire subject of the medicinal actions and uses of hemlock requires to be investigated anew; and it well deserves investigation, considering its singular energy and peculiar effects as a poison." 364.

We have transcribed nearly the whole of Dr. Christison's remarks on the actions and uses—we have only to add the well-known caution with regard to *conium*—take care that the preparations do actually contain conine.

The same complete method of description is pursued in the article on *Sulphate Magnesia*.

" MAGNESIÆ SULPHAS, *E. L. D.* *Sulphate of Magnesia.*

" Tests, *Edin.* Ten grains dissolved in a fluid-ounce of water, and treated with solution of carbonate of ammonia, are not entirely precipitated by 280 minims of solution of phosphate of soda (See *Tests.* 1 salt, 20 water.)

" Tests, *Lond.* Crystalline: very easily soluble: the solution does not give off hydrochloric acid on the addition of sulphuric acid. One hundred grains dissolved and added to a boiling solution of carbonate of soda yield 34 grains of dried carbonate of magnesia.

" Process, *Dub.* Take of
Commercial sulphuric acid, twenty-five parts;
Water, one hundred parts;
Carbonate of magnesia, twenty-four parts or a sufficiency.
Mix the acid and water, and gradually add the carbonate to saturation.
Evaporate the filtered liquor, so that it may crystallize on cooling.

" For. Names.—*Fren.* Sulphate de Magnésie; Sel d'Epsom.—*Ital.* Solfato di magnesia; Sale d'Inghilterra; Sale d'Epsom.—*Span.* Sal. amarga; Sal. de la higuera.—*Port.* Sal. cathartico amargo.—*Ger.* Schwelfelsaure magnesia;

Bittersalz; Ebsamer-salz: Seidlitzer-salz.—*Swed.* Bittersalt; Engelskt-salt. *Dan.* Engelsk laxeersalt.—*Russ.* Sernokislaia magnesia." 615.

Its *General and Natural History* is soon discussed.

"SULPHATE OF MAGNESIA (Epsom salt, English salt, Bitter salt, Seidlitz salt,) was discovered in 1694 by Grew (Geiger). It exists abundantly in some mineral springs, as in those of Seidlitz and Saidschütz* in Bohemia, of which it constitutes about one per cent, and above one-half the total saline contents. In the water of Epsom, whence it has derived its familiar name, it amounts to about a four-hundredth of the water. It is also contained in considerable proportion in the bittern of sea-water, from which common salt has been prepared. It is farther found in some soils, and sometimes effloresces in capillary crystals." 615.

*Chemical History.*—The various methods of obtaining the salt and its chemical characters are sufficiently fully detailed. The most important methods are the following.

"From bittern alone it may be obtained by simple evaporation and crystallization; but it is also usual in the first place to add a little sulphuric acid to the liquid, so as to convert into sulphate the chloride of magnesium which forms part of the saline ingredients." 615.

We do not recommend this process. The chlorides are scarcely ever entirely got rid of, and a more or less deliquescent salt is apt to be obtained. The several processes of Dr. Henry of Manchester, which succeed, are much better, more especially the following:

"From dolomite, or magnesian limestone,—a common mineral, composed of carbonate of lime and carbonate of magnesia,—it may be prepared by converting the carbonates into sulphates, and separating the sulphates by means of the inferior solubility of that formed with lime." 615.

Amongst the chemical characters, which are too familiar to require further mention, sulphate magnesia is said "when slowly crystallized, to form large rhombic prisms, often truncated on the obtuse edges, and terminated by two or four converging planes. Sulphate of zinc and sulphate of soda have the same crystalline form." Surely sulphate soda belongs to the *oblique* prismatic system.

"The sulphate of magnesia *now* used in Britain, seldom contains any impurity." For proving that it really contains no base but magnesia, Dr. Christison prefers the tests of the Edinburgh Pharmacopœia to the London tests; and the method must undoubtedly be more simple, and more easily performed.

*Actions and Uses* are known to most persons, but Dr. Christison mentions a fact perhaps not so generally known—the effect of the addition of sulphuric acid. He says:—"Sulphuric acid is an important addition. When added in sufficient quantity it covers the bitter taste of the salt, makes it

---

* To which we may add Pülna. Indeed, from the account of Mr. Spitta, in Dr. Johnson's "Pilgrimages to the Spas," it would appear to contain the largest quantity. Singularly enough, too, on the same authority, the spring at Sedlitz is quite unemployed, and has been so for 33 years.

sit easier on the stomach, counteracts its refrigerant effect, does not at all impair its energy, removes altogether its tendency to gripe or irritate the rectum, and even prevents it from interfering with the appetite and digestion."

Sufficient, we hope, has been said to set forth with clearness the plan and scope of the present volume. The works of Dr. Christison stand in no need of public recommendation.

---

### IV. Elements of Materia Medica and Therapeutics.

The first edition of this very valuable work appeared in 1840, and we doubt not that many of our readers are acquainted with it. We are not, in general, in the habit of reviewing second editions; but the present instance, we have much pleasure in making an exception. To those who are familiar with Dr. Pereira's first edition, we shall first address a few remarks, and state the points of difference between it and the present; after which we shall proceed to the general review of the work, as usual.

The principal additions to the present edition are the articles on, what Dr. Pereira terms, the Psychical Agents, including the Mental Impressions considered as therapeutical agents; and the articles on Light, Heat, Electricity, Magnetism, and the Hygienic Agents, Diet, Exercise, and Climate. The effects of light and heat, in all their modes of application to the body, are examined. A distinct section is given to electricity of tension, galvanism, and to magnetism. The article on food includes some of the recent researches of Dr. Prout on the subject of assimilation, into which we entered fully in a late review. Dr. Pereira adopts Dr. Prout's division of *simple* alimentary substances, into the saccharine, oleaginous, and albuminous; and treats of them separately under these heads. The *compound* aliments are said to be taken from the animal or vegetable kingdom. The relative merits of fish, flesh, fowl, and vegetable, in a therapeutical point of view, are discussed fully, and an interesting table of the diets of the different London hospitals is subjoined. But besides the introduction of these new articles into this edition, the processes of the British Pharmacopœias (including those of the new Edinburgh one) have been described in a more detailed maner. Upwards of a hundred woodcuts have also been added, which very much increase the interest of the work. They comprise figures of crystals, and of some insects used in medicine; microscopic views of the amylaceous grains of commerce, for which we are to tender our thanks to Mrs. Pereira; illustrations of chemical manufactures, and of the modes of preparing some vegetable products. The text also has been disencumbered of the references to different works, which have been thrown into the form of foot-notes, and in the typographical part, various improvements have been effected. These additions have of course increased the size of the volume, but not to any great extent.

We shall now proceed, without further preliminary observations, to our review.

The object of our author in this publication is more extended than appears at the first inspection of his title-page. He proposes to give, not only a description of the materia medica, and their application to the cure of disease; but to include a concise account of the most important modern discoveries in natural history, chemistry, physiology, and therapeutics, as far as they pertain to pharmacology. It is with this view that woodcuts of the different animals used in medicine are introduced, and woodcuts of the principal chemical manufactures of the different compound articles of the materia medica. And it is with this view, we doubt not, that the recent experiments of physiologists on the action of different medicines, are detailed at considerable length. With regard to the best arrangement of the materia medica; it is still open to enquiry. Dr. Pereira has discussed this point at some length in his introductory remarks on general therapeutics; and he considers the natural-historical as the best, and has adopted it in the present work. We cannot doubt the practical superiority of the therapeutical arrangement, provided it *could* be carried out. But who is there so bold as to assign unquestionable places in a therapeutical arrangement, for all the materia medica. We need only quote one instance. Where would mercury be put? It is a stimulant, a sedative, a tonic, an alterative, a sialogogue, a specific, according to the varying views of the authors who have written on it. Next to the therapeutical, which cannot be, or cannot, at least be perfect, we prefer, with Dr. Pereira, the natural-historical. Our readers are now to be informed of the general plan of this arrangement. The first grand natural division of remedies is into *Organic* and *Inorganic*. The former includes the non-metallic and metallic elements, and their compounds: the latter, the vegetable and animal products. The physico-chemical history and uses of the former occupy the first volume. The vegetables are arranged according to the natural system of De Candolle and Lindley, and each drug from the vegetable kingdom will be found under the natural order of the plant which yields it.

The animals are classed according to the system of Cuvier.

The difficulty of finding any substance has been urged, we are aware, against the present arrangement; but this is a difficulty in imagination only: for the index of the present work, which is most elaborate and accurate, will entirely obviate it.

Our readers are thus acquainted with the general plan of this work; and they should now be informed that, previous to the special history of the materia medica, there is an Introduction of great length on the general properties of medicines. This was necessary to render the subject complete. We shall proceed to analyse this Introduction, and then give some examples of the method pursued by Dr. Pereira in the description of the materia medica individually.

We are led in Chap. 2, to assign all the effects of medicines to three acting forces,—mechanical, chemical, or dynamical force—the last term being applied to all actions not strictly referrible to the two first; and from the operation of one, two, or all of these forces in a medicine, the effects considered physiologically will be either local or remote; local from their primary action on the part to which they are applied, remote from their influence on the system. Of the local effects we need say but little, they may be expected *à priori* from our knowledge of the physico-chemical properties of the medicine; but the production of the remote effects is not so easily explained. Absorption and sympathy are the two rival doctrines on this point, and Dr. Pereira devotes a chapter to the consideration of each.

The Chapter on Absorption is well worth perusal. Dr. Pereira first establishes the point that poisons *are* absorbed. He proves this by these two simple facts, viz. "the disappearance of certain substances from a shut cavity into which they had been introduced, and the detection of medicinal particles in the blood, secretions, or solids of the body."

With regard to the vessels effecting absorption, Dr. Pereira continues: "The particles of medicinal and poisonous substances are absorbed by the veins principally, but also by the lymphatics and lacteals."

And he establishes his positions by the following remarks.

"1. Absorption by the Veins.—The circumstances which seem to prove venous absorption are the following:—

"*a. Detection of substances in the venous blood.*—Tiedemann and Gmelin administered a variety of colouring, odorous, and saline substances to animals, mixed with their food, and afterwards examined the state of the chyle, and of the blood of the (splenic, mesenteric, and portal) veins. The *colouring* substances employed were—indigo, madder, rhubarb, cochineal, litmus, alkanet, gamboge, and sap-green; none of them could be detected in the chyle, but some were found in the blood and urine. The *odorous* substances used were—camphor, musk, spirits of wine, oil of turpentine, Dippel's oil, asafœtida, and garlic: they were, for the most part, detected in the blood and urine, but none were found in the chyle. The *saline* substances tried were—acetate of lead, acetate and cyanuret of mercury, chloruret and sulphate of iron, chloruret of barium, and ferro-cyanide and sulpho-cyanide of potassium. A few of these were detected in the chyle, and most of them in the venous blood and urine. From these experiments we may conclude that, although saline substances occasionally pass into the chyle, odorous and colouring matters do not; all the three classes of substances, however, are found in the venous blood. These results, observe Tiedemann and Gmelin, are opposed to those of Lister, Musgrave, J. Hunter, Haller, Viridet, and Mattei, but agree with those of Hallé, Dumas, Magendie, and Flandrin.

"*b. Division of all Parts but Blood-vessels.—Magendie's Experiment.*—Magendie and Delille performed a striking experiment, with the view of settling, if possible, the question of venous or lymphatic absorption of medicines and poisons. They divided all the parts of one of the posterior extremities of a dog, except the artery and vein, the former being left entire, for the purpose of preserving the life of the limb. A portion of the *upas tieuté* was then applied to a wound in the foot: in the short space of four minutes the effects of the poison were evident, and in ten minutes death took place. To the inferences drawn from this experiment, however, several objections have been stated: first, the exhibition of opium, to diminish the pain of the operation, has been said to vitiate the whole of the experiment; secondly, the coats of the arteries and veins contain lymphatics, by which absorption might be carried on; and thirdly, as the poison was introduced into a wound, the poison might have combined with the blood, and have rendered it deleterious, without the process of absorption taking place. The first two of these objections have been obviated. In a second experiment, Magendie severed the artery and the vein, and reconnected them by quills, so as to preclude the possibility of absorption taking place by the lymphatics of these vessels: the effects were the same. Some years since I assisted my friend Mr. Lloyd, assistant-surgeon of St. Bartholomew's Hospital, in performing an analogous experiment, using *strychnia* instead of the *upas tieuté*, and without administering opium: death took place in twelve minutes.

"The late Dr. Thomas Davies observes, on this experiment, that as the absorbents and veins communicate, it is possible that the poison flowed first into the absorbents, and from thence into the veins of the amputated portion of the limb.

" *c. Lacteals tied: effects of poisons still produced.*—Magendie says that symptoms of poisoning were observed in six minutes, when nux vomica was applied to the intestine, though the lacteals had been tied.

" *d. Bloodvessels tied: poisons do not act.*—Segalus tied the veins of a portion of intestine, and applied poison, but no effects were produced. Emmert observed, that when the abdominal aorta was tied, hydrocyanic acid was applied to the foot without producing any effect, but when the ligature was removed, symptoms of poisoning came on. Mr. Blake found that if a ligature be put around the vena portæ, and then poison be introduced into the stomach, it failed to act.

" It deserves notice, that the Academy of Medicine of Philadelphia found that nux vomica, introduced into the intestines, produced tetanus, although the vena portæ was tied.

" *e. Rapidity of absorption and circulation too great for the lymphatics or lacteals.*—Mayer found that ferro-cyanide of potassium could be detected in the blood, in from two to five minutes after its injection into the lungs. From this it has been inferred that it enters the blood too speedily for it to be explained by the slow circulation of the lymph. From later experiments, it appears that the rapidity with which poisons enter the blood has been greatly underrated. Professor Hering, of Stuttgardt, found that the time which a solution of ferrocyanide of potassium, injected into the jugular vein, required to reach that of the opposite side, was in various experiments, from twenty to thirty seconds. And Mr. Blake states that the time required for a substance which does not act on the capillary tissue, to pass from any part of the vascular system back to the same part again, in dogs, varies from twelve to twenty seconds.

" Rapid as is the circulation of poisonous molecules, it has been supposed not to be sufficiently so to explain the operation of certain poisons which have been said to act instantaneously; and hence an argument has been raised in favour of the nerves being the medium by which the deadly impression is conveyed. But Mr. Blake asserts that an interval, always more than nine seconds, elapses between the introduction of a poison into the capillaries or veins, and the first symptom of its action;—a period sufficiently long foı a poison to be brought into general contact with the tissues it affects.

" 2. Absorption by the Lacteals and Lymphatics.—The particles of medicinal and poisonous substances are probably absorbed by the lacteal and lymphatic vessels, as well as by the veins. But the process seems to be slow, and, moreover, is confined to certain agents. Tiedemann and Gmelin, whose experiments I have above referred to, were unable to recognize either colouring or odorous substances in the chyle, but occasionally detected certain salts. The absorption of saline, and non-absorption of colouring matters, have likewise been noticed by others.

" Some of the experiments performed by the Academy of Medicine, as Philadelphia, appear to be in favour of absorption being effected chiefly by the lymphatics: but they are not conclusive." 112.

The mechanism of venous absorption is now adverted to. It is dependent on the principles of imbibition or permeation of membranous bodies to fluids set forth by Dutrochets, and since made of so much importance in the explanation of certain physico-vital processes. The fact then of absorption being established, it becomes in the next place a question, whether this absorption of a medicine has any thing to do with its remote effect. For, " we are not opposed," say Messrs. Morgan and Addison, "to the theory of venous absorption, but to that theory which would associate with it the *absolute necessity* for the admission of a poison into the system," in order to the production of its constitutional effects. Dr. Pereira considers the following facts substantiate the point at issue.

1. The activity of substances injected into the blood-vessels.
2. The detection of substances in the blood.
3. The activity of medicines being promoted by the means which promote absorption, and vice versâ.
4. Magendie's well-known experiment of connecting the limb of an animal with the rest of the body by two quills placed in the great artery and vein, and finding the poison act just as readily.
5. The activity of hydrocyanic acid applied to the lower extremity the same after division of the spinal marrow at the lumbar region.
6. The inefficacy of poisons to a part, above which a ligature has been placed.

But, *how* do medicines and poisons which have entered the blood-vessels affect distant organs? This is a pertinent problem of our author, and not so easily solved. Dr. Pereira suggests three ways.

1. By modifying or altering the properties of the blood, and thereby unfitting it for carrying on the functions of the body.
2. By pervading the structure of the organ acted on.
3. By acting on the lining membrane of the blood-vessels.

These points are discussed at large, and with great ability.

The arguments in favor of the doctrine of absorption are so strong from the preceding remarks, that little is left to be said on the more popular theory of sympathy. Nevertheless this doctrine has been stoutly upheld by Messrs. Morgan and Addison, and it is principally with the view of answering these gentlemen that Chapter V., "on the Operation of Medicines by Nervous Energy," has been introduced. The whole chapter has to prove or disprove the following proposition,—"that all poisons, and perhaps, indeed, all agents, influence the brain and general system, through an impression made upon the sentient extremities of the nerves, and not by absorption and direct application to the brain."

Dr. Pereira enumerates the four following circumstances, as having been adduced in *favor* of this view, and proceeds to comment on each.

"1. The rapid action of some poisons.
2. The effects being disproportionate to the facility for absorption.
3. The effects of several poisons being analogous to those of severe injuries.
4. The rapidity of action not being diminished by increasing the distance of the brain from the part of the vascular system into which the poison is introduced." 123.

We cannot curtail the subsequent observations.

1. *The rapid Operation of some Poisons.*—One drop of pure hydrocyanic acid, says Magendie, placed in the throat of the most vigorous dog, causes it to fall dead after two or three hurried inspirations. Sir Benjamin Brodie once happened to touch his tongue with the end of a glass rod which had been dipped in the essential oil of bitter almonds; scarcely had he done so, before he felt an uneasy, indescribable sensation at the pit of the stomach, great feebleness of limbs, and loss of power to direct the muscles, so that he could hardly keep himself from falling. These sensations were quite momentary. In the cases now quoted, the rapid action of the poisons seems almost incompatible with the idea of their absorption. Dr. Christison says, that two grains of conia, neutralized with thirty drops of diluted muriatic acid, being injected into the femoral vein of a young dog, stopped respiration, and with it all external signs of life, in two seconds, or three at farthest.

"In this case the death appears to have been too speedy to admit of the supposition that the effect occurred in consequence of the direct contact of the poison with the brain or spinal-marrow.

"Mr. Blake has met this argument by declaring that poisons are not instantaneous in their action, but that sufficient time always elapses between the application of a poison and the first symptom of its action, to admit of its contact with the tissue which it affects. Thus he found that, after half a drachm of concentrated hydrocyanic acid had been poured on the tongue, eleven seconds elapsed before any morbid symptom appeared, and death did not occur until thirty-three seconds after the exhibition of the poison; and on repeating Dr. Christison's experiment, he found that fifteen seconds elapsed after ten drops of conia (saturated with hydrochloric acid) had been injected into the femoral vein of a dog, before symptoms of the action of the poison appeared; and death did not occur until thirty seconds after the injection. Now the time required for a substance to be absorbed by the capillaries, and diffused through the body may not exceed, according to Mr. Blake, nine seconds. So that the interval which elapsed in the preceding experiments, between the application and the effect, is quite sufficient to admit of the absorption and circulation of the poison.

"2. *The effects being disproportionate to the facility for absorption.*—Orfila says that alcohol acts with much less energy when injected into the cellular texture, than when taken into the stomach; and, as the power of absorption is greater in the former than in the latter part, he concludes that the remote action of alcohol must, in the first instance, be produced by the agency of the nerves. Opium, on the contrary, is supposed to operate by absorption, because it is more active when injected into the cellular texture than when taken into the stomach.

"This experiment requires repetition. Even if the result be as stated by Orfila, the inference drawn from it is by no means a necessary one. As alcohol coagulates the blood when mixed with this fluid, its absorption would be more active in the dilute than in the concentrated state. Now, the secretions and contents of the stomach may, by diluting the alcohol, promote its absorption.

"3. *The effects of some poisons being analogous to those of severe injuries.*—Thus, a tetanic state is produced by strychnia as well as by a punctured wound.

"As tetanus can be produced without the absorption of any thing (as when it arises from the laceration of a nerve), it is not necessary, it has been urged, to suppose this process in the case of strychnia. Mr. Blake has endeavoured to meet this objection by suggesting that, in the first case, the disease may arise from the propagation of some pathological state from the injured nerve to the nervous centres; for were the symptoms the mere result of the local irritation of a nerve, we might expect, he observes, to produce them at pleasure, by merely irritating the nerve; but it is well known, he adds, that this is not the case. The latter part of this statement is not quite correct; for Dr. Marshall Hall says, that 'if one of the lateral nerves [of the decapitated turtle] be laid bare, and pinched continuously, the muscles of the upper extremities, as well as the lower, are forcibly contracted. This is, in my opinion, the very type of tetanus.'

"4. *The rapidity of action not being diminished, by increasing the distance of the brain from the part of the vascular system into which the poison is introduced.*—Messrs. Morgan and Addison found, that the woorara poison produced its effects, when thrown into the femoral artery, a few seconds sooner, than when introduced into the carotid artery. Now, if contact with the brain were necessary to the action of this poison, a longer time would be required in the former than in the latter case for the production of any morbid symptoms.

"Mr. Blake, however, asserts that in his experiments be found, that the nearer to the nervous centres is the part of the vascular system into which the poison is introduced, the more rapid is its action." 125.

Chap. VI. alludes to the parts affected by the remote action of medicines; and, in a practical point of view, this seems most important; for whether a remedy act by sympathy or by absorption, whether it alter the condition of the blood or pervade the structure of the organ—the *how,* in fact, it produces its effects, is, after all, of little moment to the physician, compared with the practical knowledge of the organ on which it uniformly acts. We have said Dr. Pereira *alludes* to the parts affected; for, in this chapter, he merely enumerates the different parts on which different medicines act. Mercury, for example, and alkalis he considers to act on the blood—opium, green tea, and alcohol produce their effects on the cerebrum—foxglove and tobacco diminish the force of the circulation—and so on.

The introduction however is not yet nearly completed. Dr. Pereira mentions in Chap. VIII. the circumstances both with reference to the medicine itself—its state of aggregation, chemical and pharmaceutical mixture —and with reference to the patient, sex, age, habit, and more especially his disease, which modify the actions of medicines, and he comments in Chap. X. on the different parts to which medicines are applied.

The last Chapter, XII., supplies the place of the useful remarks found under each head in works on materia medica, with the therapeutical classification. Dr. Pereira here defines and illustrates all the important therapeutical terms so continually employed, in reference to the actions of medicines, therapeutically considered. We need not bring these remarks before our readers. We shall only mention one new term made use of by our author. Dr. Pereira applies the term *liquefacient* to a subclass of evacuants, and thus defines it.

"Medicinal agents which augment the secretions, check the solidifying, but promote the liquifying, processes of the animal economy, and which, by continued use, create great disorder in the functions of assimilation, may be termed *liquefacients* (from *liquefacio,* I liquify)." 194.

*Mercury, Antimony, Iodine, and the Alkalis* are examples. The term, in fact, approaches in signification to our terms resolvent, alterative, or perhaps to the two combined. We leave it with the public to judge as to the necessity of its introduction.

And now we shall present to our readers two examples of the method pursued by Dr. Pereira, in the description of the individual articles of the Materia Medica.

*Acidum Hydrocyanicum Dilutum.*—This acid is a compound of carbon, nitrogen, and hydrogen. Dr. P. has already described these elements individually, and he has treated of the compounds of carbon and nitrogen with hydrogen. We are now introduced to the compound of carbon and nitrogen forming cyanogen, and to prussic acid.

"History.—The substance called *Prussian* or *Berlin blue* (*Cæruleum Borussicum* seu *Berolinense*) was accidentally discovered by Diesbach at the commencement of the 18th century, and various conjectures were soon offered regarding its nature. In 1746, Dr. Brown Langrish published some experiments made with laurel-water in order to investigate its effects on animals. In 1752, Macquer announced that Prussian blue was a compound of oxide of iron, and some colouring principle which he could not isolate; and in 1772, Guyton Morveau concluded that this principle was of an acid nature. Scheele, in 1782, removed some of the mystery connected with Prussian blue, by obtaining *hydrous prussic acid* from it. In 1787, Berthollet ascertained this acid to be a compound of carbon, nitrogen, and hydrogen. In 1800 and 1802, Bohn and Schrader discovered it in laurel-water. Borda, Brugnatelli, and Rasori, first employed the acid in medicine, from 1801 to 1806. In 1815, Gay-Lussac obtained the acid in its pure *anhydrous* state, and explained its composition.

"SYNONYMES AND ETYMOLOGY.—It has been denominated *Prussic (Acidum Borussicum), Zootic (Acidum Zooticum),* or *Hydrocyanic Acid:* the first name indicates the substance (Prussian blue) from which it was obtained, the second refers to its animal origin, and the third indicates its constituents, hydrogen and cyanogen (so called from κύανος, *blue;* and γεννάω, *to produce;* because it is one of the constituents of Prussian blue).

"NATURAL HISTORY.—Hydrocyanic acid is a product peculiar to the organised kingdom. It may be readily procured from many *vegetables*, more especially those belonging to the sub-orders *Amygdaleæ* and *Pomeæ*: as from Bitter Almonds, Apple-pips, the Kernels of Peaches, Apricots, Cherries, Plums, and Damsons; the Flowers of the Peach, Cherry-laurel, and Bird-cherry; the Bark of the latter, and the root of the mountain ash. It is said to have been also obtained from plants of other families, as from *Rhamnus Frangula* and Ergot of Rye. In some of the vegetables now referred to, hydrocyanic acid does not exist ready formed, but is a product of the process by which it is obtained. This has been fully proved in the case of the bitter almond, and is inferred in other instances.

"This acid is rarely, if ever, found in animals. One of its constituents (cyanogen) has, however, been detected, in combination with iron, (forming Prussian blue) in the urine, the menstrual fluid, and the sweat: and with sulphur and potassium in the saliva. The greenish-blue discharge of some ulcers probably depends on the presence of Prussian blue. In one case I detected the presence of iron in this discharge. During the decomposition of animal matters, cyanogen is frequently generated: as when blood and carbonate of potash are calcined in an iron pot. It has also been stated, that when cheese is exposed to the action of water and the sun, it disengages ammonia, and if treated, in this state, by alcohol, yields traces of hydrocyanic acid." 429.

The Preparation of the acid now follows. Dr. Pereira subjoins the four methods in ordinary use for obtaining it. The London and Edinburgh Colleges make it by the action of diluted sulphuric acid on ferrocyanide of potassium.

The re-actions of the elements of these ingredients are complicated, but the explanation is very much facilitated by the aid of the skeleton diagrams now so commonly used in works on chemistry. Dr. Pereira explains all his decompositions with these diagrams.

The second method (proposed originally by Mr. Everitt) is also directed in the London Pharmacopœia, viz. the action of hydrochloric acid on cyanide of silver.

The third method is exactly similar in principle to the second; but the bicyanuret of mercury is substituted for the cyanide of silver, and the bichloride of mercury is left instead of the chloride of silver.

The fourth method proposed by Dr. Clarke, and adopted by Mr.

Laming, is the action of *tartaric acid* on *cyanide of potassium*—we have no experience of this; but we should conceive, with Dr. Pereira, that "the trouble and expense of procuring pure cyanide of potassium, and the liability of the salt to undergo spontaneous decomposition," were very cogent objections.

Dr. Pereira now adds the *properties* of the pure and dilute acid.

"PROPERTIES. *α. Of Anhydrous Hydrocyanic Acid.*—Anhydrous hydrocyanic acid is a solid at 0° F. (some state at 5° F.), having then the appearance of crystallized nitrate of ammonia; it readily melts, forming a limpid, colourless liquid, with an intense and peculiar odour; its taste is at first cool, then hot; at 45° its sp. gr. is 0·7058, and at 64½ is 0·6969. In this state it is exceedingly volatile: a drop placed on paper freezes by its own evaporation. It unites with water and alcohol in every proportion. At 79° or 80° F. it boils, forming hydrocyanic acid vapour, which is combustible; and when mixed with oxygen, explodes. Two volumes of the vapour require two and a half volumes of oxygen gas for their complete combustion. The products are two volumes of carbonic acid gas, one volume of nitrogen, and one volume of aqueous vapour." 432.

"Anhydrous hydrocyanic acid undergoes speedy decomposition. Yet Dr. Christison says he has kept it unchanged for a fortnight in ice-cold water.

"*β Of Diluted Hydrocyanic Acid.*—Diluted or medicinal hydrocyanic acid is a colourless, transparent liquid, having the taste and smell of the strong acid, but in a lesser degree. Heated in a tube it gives off a combustible vapour."* 432.

The Composition of the pure acid is now stated, and the Strength of the diluted acid, according to the London, Edinburgh, and Dublin standards; and adds, justly enough, "the discrepancy in the strength of the acid ordered in the British Pharmacopœias, is greatly to be regretted. Most of the acid met with in the shops of London chemists, is stated by the label to be of 'Scheele's strength.' But as Scheele's process gave an acid of variable strength, this statement is by no means definite. A manufacturer of large quantities of the acid informs me he sells, under the name of Scheele's acid, a diluted hydrocyanic acid, which contains 4 per cent. real acid."

"PURITY.—Diluted hydrocyanic acid should be perfectly colourless. Decomposed acid is frequently, but not invariably, coloured. It should be vaporizeable by heat: this character shows the absence of fixed impurities. The presence of metallic matter is recognised by hydrosulphuric acid, which has no effect on the pure acid. If the acid strongly redden litmus, it must contain some other acid, most probably the sulphuric or hydrochloric. The presence of any foreign acid is easily determined by the *hydrargyro-iodo-cyanide of potassium.* This salt is easily formed by adding a concentrated solution of bicyanide of mercury to a solution of iodide of potassium; a precipitate of white or pearly crystalline plates of this salt is immediately formed. If a small portion of this salt be placed in diluted hydrocyanic acid, no change is observed unless some foreign acid be present; in the latter event the red biniodide of mercury immediately makes its appearance. For this test we are indebted to Dr. Geoghegan. Sulphuric acid may be detected by a solution of the salts of barium. 'Solution of nitrate of baryta occasions no precipitate' in the pure acid *(Ph. Ed.)*; but if

* An appropriate skeleton diagram here illustrates its combustion with oxygen.

sulphuric acid be present, it occasions a white precipitate (*sulphate of baryta*), insoluble in nitric acid. Hydrochloric acid is recognized by nitrate of silver, which forms therewith white chloride of silver insoluble in boiling nitric acid, whereas the white cyanide of silver is soluble in nitric acid at a boiling temperature. I would observe, that the presence of either of these acids is no further objectionable, than that it creates a difficulty in the determination of the strength of the hydrocyanic acid: while, on the other hand, it confers the advantage of rendering the hydrocyanic acid much less liable to decompose. The acid prepared from ferrocyanide of potassium will keep for years (Dr. Christison has had some unchanged for two years and a half, though it was exposed to day-light), owing, it is supposed, to the presence of some sulphuric acid. Mr. Barry adds a little hydrochloric acid to all his medicinal hydrocyanic acid, in order to preserve it. As air and light hasten, though they are not essential to, the decomposition of the acid, they should be carefully excluded." 434.

Dr. Pereira now gives the tests for prussic acid; and the method of its detection in cases of poisoning. To the four tests—*odor*—*nitrate silver* and *boiling nitric acid*—*sulphate of copper, potash,* and *hydrochloric acid, sulphate of iron, potash,* and *hydrochloric acid,* Dr. Pereira adds another, *tincture of guaiacum* and *sulphate of copper.* We should not be inclined to set much value on this last. With regard to the Prussian blue test, it is conducted thus:

"Add sufficient caustic potash to the suspected acid to saturate it; then a solution of some proto- and sesqui-salt of iron: the common sulphate of iron of the shops, or the tincture of the chloride, answers very well, since both these preparations usually contain the two (*prot-* and *sesqui-*) salts of iron. A precipitate is thus obtained, which is liable to considerable variation in its colour, depending on the quantity of potash and the quality of the ferruginous salt employed; it may be yellowish brown, or greenish, or bluish. Then add dilute sulphuric or hydrochloric acid, when Prussian blue (ferrosesquicyanide of iron) will immediately mak its appearance, if hydrocyanic acid were present." 435.

Dr. Pereira continues:—

"The formation of Prussian blue is thus accounted for. When potash is added to hydrocyanic acid, water and cyanide of potassium are generated. By the re-action of this salt on a proto-salt of iron the proto-cyanide of iron is produced, while with a sesqui-salt of iron it forms sesquicyanide of iron. The two ferruginous cyanides, by their union, constitute the ferrosesquicyanide of Prussian blue." 435.

The action of the mineral acid might as well have been added. It re-dissolves the oxyde of iron thrown down by the potash; and thus, on the one hand, exhibits the blue color of the ferrosesquicyanuret well-marked, when *hydrocyanic acid* is present; on the other hand, renders the solution perfectly clear, if the acid have been absent.

After shewing the process for detecting the poison in organic mixtures, we arrive at the *Physiological Effects of Prussic Acid* on the organic kingdom.

In illustration of its poisonous action *on vegetables*, Dr. Pereira instances the facts of the stamens of the *Berberis Vulgaris*, and the leaves of the *Mimosa Pudica*, losing their irritability when their stems are immersed in the diluted acid.

In the animal kingdom—

"Experiments have been made with it on the following:—*Mammalia, Aves,*

*Reptilia, Amphibia, Pisces, Gasteropoda, Annelida, Crustacea, Insecta,* and *Infusoria.* The general effects are very similar on all classes, and consist essentially of loss of sensation and voluntary motion, with convulsive movements." 437.

He now considers its action on man.

"*αα. In small or medicinal doses.*—Small doses of hydrocyanic acid sometimes relieve certain morbid conditions (as of the stomach), without producing any remarkable alteration in the condition of the general system. If the dose be cautiously increased, and its operation carefully watched, the following effects are usually observed:—a bitter but peculiar taste; increased secretion of saliva; irritation in the throat: frequently nausea; disordered and laborious respiration (sometimes quick, at others slow and deep;) pain in the head, giddiness, obscured vision, and sleepiness. The vascular system is in some cases not obviously, but in others much, affected, though not uniformly; its action being sometimes quickened, at others reduced in frequency. In some instances faintness is experienced. Drs. Macleod and Granville have noticed salivation and ulceration of the mouth during its medicinal use.

"*ββ. In poisonous doses: convulsions and insensibility* (Epilepsy?): *if death occur, it takes place slowly.*—Immediately after swallowing the acid, a remarkably bitter taste is experienced; this is soon followed by a sensation of faintness and giddiness, with salivation, and is succeeded by tetanic convulsions and insensibility; the respiration is difficult and spasmodic; the odour of hydrocyanic acid may be recognised in the breath; the pupils are usually dilated, though sometimes contracted; the pulse is small or imperceptible. When recovery takes place it is usually very rapid, and the whole period of suffering seldom exceeds half an hour." 438.

"*γγ. In poisonous doses: death rapid with or without convulsions.*—In these cases the death is so rapid that, in the human subject, the symptoms have scarcely been observed. They are probably similar to those noticed in animals,—viz. imperceptible pulse, breathing not obvious, or there may be two or three deep, hurried inspirations, insensibility, and death. Convulsions may or may not be present." 438.

After a little discussion on the two important medico-legal questions connected with the action of this acid—namely, the time at which it begins to operate, and the period in which it proves fatal, we are brought at once to the *post-mortem appearances.* They are,

"Glistening and staring expression of the eyes, but which, however, is not a constant phenomenon, since it was not observed in the seven Parisian epileptics: nor is it peculiar to this poison, for the same is observed after death by carbonic acid, and in other cases (Christison): the odour of the acid is oftentimes very obvious in the blood, brain, chest, or stomach: the venous system is usually gorged with blood, while the arteries are empty: the blood is, in many cases, fluid, dark, or bluish black, and viscid or oily: the vessels of the brain and spinal-marrow are frequently gorged with blood; and the cerebral ventricles sometimes contain a serous or sanguineous liquor; the lungs are, in some instances, natural—in others, turgid with blood: the internal lining of the stomach is sometimes red." 439.

Dr. Pereira adds:

"I have examined a considerable number of animals (principally rabbits) destroyed by hydrocyanic acid, and have always found the muscles to be powerfully affected by the galvanic influence: nor have I once met with a single case in which the heart had ceased to beat when the chest had been laid open immediately after death." 439.

This last fact seems of great moment. It shews clearly that Prussic acid does not palsy the heart; and argues much in favour of its destroying at once the *vitality* of the blood. Our author, however, will speak fully of its action in his next point of enquiry—the *modus operandi.* We shall not do him justice without transcribing the whole section.

" MODUS OPERANDI.—There are several interesting subjects of inquiry connected with the operation of hydrocyanic acid, which, as they are principally theoretical, I shall briefly notice under this head.

" *a. Local Action.*—Dr. Christison says that Robiquet's fingers became affected with numbness, which lasted several days, in consequence of their exposure for some time to the vapour of this acid. This effect would appear to depend on the local action of the poison on the nerves,—a mode of operation which we are constrained likewise to admit in the case of some other narcotics. The alleviation of gastrodynia by hydrocyanic acid depends probably on this benumbing effect. Some of the local effects produced by hydrocyanic acid are those of an irritant: such are, the acrid impression made by the vapour on the nose and mouth—the ptyalism—the vomiting and purging—and the redness of the mucous membrane of the stomach.

" *β. Absorption.*—That hydrocyanic acid becomes absorbed, is proved by its having been detected by Krimer (quoted by Dr. Christison, p. 15), in the blood of animals poisoned with it, and by the odour of it exhaled by various parts of the body. The exhalation by the breath of the odour of the acid may sometimes serve to recognise the presence of the poison in the system.

" *γ. Are the remote effects of this acid caused by its absorption?*—In many cases the operation of hydrocyanic acid on the system is so rapid, and death so speedily follows the application of the poison, that doubt has been entertained of its action being dependent on its absorption. The principal arguments which have been adduced in favour of the agency of absorption are the following:—*first,* that the acid produces no remote effects when applied either to the nerves or brain: *secondly,* that applied to the tongue or stomach, it operates as an energetic poison, although the nerves of these parts were previously divided: *thirdly,* that if the acid be applied to a part where circulation is arrested, the operation of the poison is prevented: *fourthly,* the activity of the acid is in proportion to the absorbing powers of the part with which it is placed in contact: *fifthly,* a sufficient time always elapses between its application to the body, and the first symptom of its action, to admit of its operation by absorption.

" *δ. Organs affected.*—The parts specifically affected by this acid are the brain and true spinal system. The pain in the head, the insensibility, and the coma, are evidence of the cerebral affection; while the tetanic convulsions depend on the disorder of the true spinal system. Marx mentions the following experiment performed by Wedemeyer and which shows the independent action of the acid on the spinal marrow: the spinal cord of a dog was divided between the last dorsal and first lumbar vertebræ, so that the hind legs were completely paralyzed and insensible to mechanical irritants: hydrocyanic acid was then introduced into one of the hind legs;—in one minute, symptoms of poisoning commenced, the hind as well as the fore legs were violently convulsed,—and in twelve minutes the animal was dead. The affection of the respiratory and circulatory system produced by hydrocyanic acid is probably only secondary: that is, is the result of the influence of this agent over those parts of the nervous system from which the respiratory organs and heart derive their nervous power. The insensibility caused by hydrocyanic acid occurs too rapidly, in many cases, to be the result of asphyxia caused by paralysis of the muscles of respiration.

"ε. *Condition of the brain and spinal marrow induced by this acid.*—The precise pathological condition of the brain and spinal cord of an animal under the influence of hydrocyanic acid, cannot be positively determined, and is, therefore, a matter of conjecture. Whatever it may be, it is probably identical with that which occurs during an epileptic paroxysm, and with that produced by loss of blood: for the essential symptoms (insensibility and convulsions occurring suddenly) are the same in all three states,—and ammonia has been found to relieve them. Now Dr. Hall has shown that the convulsion from hæmorrhage is spinal. Dr. Holst, Professor of Materia Medica in the University of Christiana, Norway, told me of a case of epilepsy which had been under his care, and in which it was observed that the pulse in one arm was always imperceptible during the paroxysm. On a post-mortem examination it was discovered that an anomalous distribution of the arteries existed,—so that this arm was supplied with blood by the vertebral arteries, which derived it, through the basilar artery, from the carotids. Now the cessation of the pulse during the paroxysm proved that the circulation through these vessels was temporarily interrupted. Does any similar interruption occur in poisoning by hydrocyanic acid?

"ϑ. *Cause of death.*—In most cases the immediate cause of death is obstruction of respiration. In some instances it is stoppage of the heart's action. There are cases, however, in which the death is too immediate to be produced by obstructed respiration, while, on opening the chest, the heart is found still beating: this I have observed in experiments on rabbits with strong hydrocyanic acid.

"η. *Cumulative effects.*—Hydrocyanic acid is not usually regarded as a cumulative poison; but a case mentioned by Dr. Baumgärtner (quoted by Dr. Christison), as well as some other circumstances, seem to favour the reverse opinion." 441.

*Uses of Prussic Acid.*—The first and foremost complaint for which hydrocyanic acid is prescribed, is gastrodynia, with or without pyrosis, with or without vomiting. This needs no comment. But Dr. Pereira has found it useful in a painful affection of the bowels, analogous to the stomach-affection above mentioned, which he purposes to call enterodynia.

He has also seen it used with great success to allay vomiting and purging in severe forms of ordinary English cholera, when opium has completely failed. In Asiatic or malignant cholera it has occasionally appeared to be serviceable, and he has found it successful in checking the *diarrhœa* of *phthisis*, when logwood, chalk, and opium had failed. These are its principal uses. Dr. Pereira alludes to its employment in genuine spasmodic asthma, and in hooping-cough, in the combination of Dr. Roe, with ipecacuan, and tartarized antimony, (powerful adjuncts) also in epilepsy, chorea, hysteria, tetanus, and hydrophobia, yet without any eulogiums. In cancer, tic-douloureux, and rheumatism, it is considered anodyne. As a lotion also, in impetigo, prurigo, and psoriasis, it is recommended by Dr. A. T. Thompson. As an injection in cancer of the uterus by Frisch of Nyborg, and in gonorrhœa by Schlegel, with advantage.

We pass on to our next example from the vegetable kingdom. Pursuing the natural arrangement of plants, Dr. Pereira commences with the lowest forms of vegetable existence, the Cryptogamia or flowerless plants. He then treats of Endogens, the next perfect in organization, including, besides others, the following important natural orders—Gramineæ melanthaceæ—Liliaceæ—Smilaceæ—Iridaceæ—and lastly arrives at the most perfectly-developed plants—Exogens.

These last include all the more important natural orders; and of each natural order, prior to the description of the particular plants, there is a distinct botanical description. Before describing *Senna,* for example, we have a description of the natural order *Leguminosæ,* condensed from De Candolle. Moreover, senna belongs to the sub-order mimoseæ.

After the *History of Senna,* Dr. Pereira proceeds at once to its *Botany.* He describes the genus Cassia thus :—

"*Sepals* five, scarcely united at the base, more or less unequal. *Petals* five, unequal. *Stamens* ten, free, unequal; the three lower ones longer, the four middle ones short and straight, the three upper ones with abortive anthers. *Anthers* dehiscing at the apex. *Ovary* stalked, frequently arched. *Legume,* various. *Trees, shrubs,* or *herbs.* *Leaves* simply and abruptly pinnate. *Petioles* frequently glanduliferous. *Leaflets* opposite." 1598.

Dr. Pereira enumerates six species of senna-leaves seen by him at different times; three of which, viz. those of the cassia obovata, c. acutifolia, and c. elongata are the most known.

The following is the description of the cassia acutifolia.

"2. C. ACUTIFOLIA, *Delile.* *Stem* suffruticose. *Leaves* pinnate; *petiole* glandless; *leaflets* five to seven pairs, lanceolate, acute. *Legumes* flat, elliptical, naked on both sides, somewhat bent on the upper margin (Delile).—An *undershrub,* about two feet high. *Leaves* when young slightly silky or pubescent. *Flowers* yellow, in axillary racemes, at the top of the branches. *Petals* obovate. *Legumes* somewhat swollen by the seeds. *Seeds* six or seven in each legume.—Egypt, in the valleys of the desert to the south and east of Assouan.—Collected by the Arabs, and sold by them to merchants who convey it to Cairo." 1599.

After alluding to the *commerce* of *senna,* we arrive at its *description,*

"DESCRIPTION.—Senna (*folia sennæ*) has a peculiar, agreeable, tea-like odour, and a nauseous, bitter taste. Its colour should be bright and fresh. If largely mixed with extraneous matter, if it be much broken or very dusty it should be rejected. Boiling water extracts about a third of its weight. Proof spirit yields a brown—alcohol or ether a green tincture."

Each of the different kinds of senna have a distinct description superadded. Alexandrian senna consists of the leaves, flowers, and fruits of cassia obovata, and cassia acutifolia, together with the leaves, flowers, and fruit of a plant belonging to the natural order, asclepiadaceæ, the *cynanchum argel.* The best East Indian or the Tinnevelly senna is a very fine unmixed senna composed of the leaves of the cassia elongata. To those who are accustomed, nothing is easier than to select the argel leaves, flowers, and fruits from the specimens of Alexandrian senna. The student, however, will find some woodcuts, which represent not only the common acutifolia, obovata and elongata leaves, and the legumes of these species of cassia; but also the argel leaves, flowers and fruit. There is one thing, however, we do not promise him; we do not promise that he will pick the flowers of argel from senna, in quite such regular corymbs, as is represented.

"Adulteration.—Senna is not, to the best of my belief, adulterated in this country. The leaflets of *colutea arborescens* or *bladder senna* have, on the Continent, been occasionally intermixed. They are elliptical, regular, and obtuse. Their regularity at the base would at once distinguish them from the leaflets of *cassia obovata*." 1603.

"Argel leaves, mixed with a few leaflets of *C. acutifolia*, I have known to be recently sold as *picked* or *heavy senna* at a higher price. It was done rather from ignorance than fraud." 1604.

Dr. Pereira alludes to a real adulteration practised on the Continent, the introduction of the leaf of the *coriaria myrtifolia*. He has figured it as a three-nerved leaf, with a strongly-marked midrib, the two lateral nerves disappearing towards the summit. This leaf, as far as we are aware, is unknown in the English market.

Three *analyses* of senna are now given, and these are followed by the *chemical characteristics* of this drug. It is very remarkable, if true, that *cathartine*, the purgative principle of senna, contains no *nitrogen*.

"Physiological Effects. *α. On Animals.*—In doses of five or six ounces it purges horses. Courten threw an infusion into the veins of a dog; it quickened the respiration, and caused vomiting. The animal appeared weak, was dull, and had no inclination to eat.

"*β. On Man.*—Regnandot injected half a spoonful of weak lukewarm infusion of senna into the left median vein of a young man affected with an herpetic eruption. The only effect produced was a slight temporary headache. Some days afterwards a spoonful was injected: in half an hour violent shivering and vomiting came on, which were followed by heat and purging. The febrile symptoms continued for several hours. Taken by the stomach senna acts as a sure and safe purgative. Its ill effects are nausea, griping, flatulence, and, at first, depression, afterwards excitement of the pulse. It appears to stimulate the abdominal and pelvic vessels, thereby having a tendency to promote the hemorrhoidal and menstrual discharges. It is one of the mildest of the drastic purgatives. Unlike scammony, gamboge, jalap, and most other drastics, it does not rank among poisons, even when given in large doses. It is distinguished from the saline purgatives by its stronger and more irritant operation, by the heat, gripings, and increased frequency of pulse, which attend its purgative action. From rhubarb it differs in being more powerful and irritant in its operation, in being nearly or quite devoid of any tonic operation. It acts more speedily and powerfully than aloes, and in a less marked manner on the large intestines. In its operation it appears to rank between jalap and aloes.

"The petioles and stalks possess similar properties to the leaflets. Formerly the griping quality of senna was ascribed to the stalks, but both Bergius and Schwilgué have proved the error of this notion. The legumes are much milder in their operation than the leaflets.

"Good East Indian senna is almost, if not quite, as active as the Alexandrian. Mr. Twining, after extensively trying it, declared it equal to the best he had ever seen. The obovate senna appears to be milder than the acute-leaved. The Senegal senna, before referred to, was found to possess less activity than ordinary senna. Part of the acrid and griping qualities of Alexandrian senna are referrible to the argel leaves, which, according to the observations of Rouillure, Delile, Nectoux, and Pugnet (quoted by Delile), possess greater activity than the true senna leaves. Rouillure says they purge and gripe, and are used by the

Arabs of Upper Egypt, without the addition of senna. These effects might be expected from the known properties of the asclepiadaceæ (before referred to.) ‘American senna is an efficient and safe cathartic, closely resembling the imported senna in its action, and capable of being substituted for it in all cases in which the latter is employed.’

“If infusion of senna be given to the nurse, the suckling infant becomes purged,—a satisfactory proof that the cathartic principle of senna becomes absorbed, and is thrown out of the system by the excretories. Furthermore, as purging results from the injection of infusion of senna into the veins, this cathartic would appear to exercise a specific influence over the bowels, independent of its local action on these when it is swallowed.” 1606.

The *uses* of senna are naturally deduced from its physiological effects. The article is finished with the London, Edinburgh, and Dublin pharmaceutical preparations.

In dismissing this work, we are taking leave of one, the perusal of which has afforded us the greatest satisfaction. It is full on every point, and yet not prolix. The introduction on general pharmacology is excellent; and the whole arrangement of the special history of the materia medica exceedingly well conducted. We cannot commend the work too strongly. The student of materia medica will find it an invaluable addition to his library.

